# Entertainment activities and the risk of multiple sclerosis: A Mendelian randomization analysis

**DOI:** 10.1097/MD.0000000000044981

**Published:** 2025-10-17

**Authors:** Zheng Wang, Wan Wu

**Affiliations:** a Department of Cardiothoracic Surgery, People’s Hospital of Kaizhou District, Chongqing, China; b Gastrointestinal Surgery Department, People’s Hospital of Kaizhou District, Chongqing, China.

**Keywords:** causal relationship, entertainment activities, Mendelian randomization, multiple sclerosis

## Abstract

Modifying environmental and lifestyle factors may have the potential to prevent and ameliorate multiple sclerosis. Further elucidating the etiology of multiple sclerosis and proposing actionable prevention measures are of significant importance, but establishing causality in epidemiological data can be challenging. This study employed a two-sample Mendelian randomization analysis to evaluate the causal effect of entertainment activity factors on the risk of multiple sclerosis. Publicly accessible summary statistics derived from genome-wide association studies were utilized to assess 14 modifiable forms of entertainment activities. The inverse variance weighted random effects method was used as the primary analytical approach to estimate causal effects. Additionally, MR-Egger, weighted median, and weighted mode methods were applied to assess robustness. Systematic sensitivity analyses and heterogeneity tests were conducted to verify the reliability of our findings. We found that spending time outdoors in the summer may prevent the development of multiple sclerosis (odds ratio = 0.995; 95% confidence interval 0.991–0.999; *P* = .010). In contrast, the other entertainment activities studied showed no significant causal relationship with multiple sclerosis. Our results suggest that spending time outdoors in the summer may protect against the development of multiple sclerosis, providing implications for preventive measures against the disease.

## 1. Background

Multiple sclerosis (MS) is a chronic inflammatory disease of the central nervous system that destroys the protective layer around nerves in the brain and spinal cord. MS is a chronic, inflammatory disorder of the central nervous system characterized by damage to myelin and axons. It can affect any part of the central nervous system, leading to a diverse array of symptoms and disabilities.^[1]^ A striking pathological feature of MS is the transient fluctuation or worsening of symptoms with increasing body temperature and an abnormal electric shock-like sensation in the spine or extremities when the neck is flexed.^[2,3]^ With MS primarily affecting young people, the socioeconomic impact of the disease is substantial. Research has shown that the financial burden of unemployment and productivity loss due to MS significantly surpasses the costs of health and social care.^[4]^ Although MS can be treated with medication or other complementary methods, the efficacy and risks of treatment increase exponentially. Furthermore, the disease is recurrent, and relapsing symptoms tend to be more intense.^[2]^ Further elucidation of the etiology of MS and the proposition of actionable prevention measures are crucial for MS prevention.

The fundamental causes of MS remain uncertain but are thought to involve the interaction of genetic and environmental factors. Confirmed environmental risk factors include low vitamin D levels, limited ultraviolet exposure, Epstein–Barr virus (EBV) infection, diet, and smoking. ultraviolet exposure stimulates the production of vitamin D in the skin. Low vitamin D levels and limited outdoor activity increase susceptibility to MS in individuals with certain genetic polymorphisms.^[5,6]^ While genetic factors are difficult to modify through medical interventions or personal behavior, altering these environmental and lifestyle factors holds preventive potential. Therefore, determining the causal relationship between these factors and MS is essential.

Investigating the causal relationship between entertainment activities as part of an individual’s adaptive behavior and the risk of MS is crucial for designing effective public health intervention strategies. Adaptive behavior results from dynamic interactions between the nervous system, the body, and the environment, and is manifested in a range of social, conceptual, and practical skills that reflect the individual’s need to adapt to their surroundings.^[7,8]^ Entertainment activities constitute a significant portion of adaptive behaviors in modern lifestyles, often involving pursuits aimed at relaxation, pleasure, or excitement, and are characterized by their practicality. Previous studies have shown that encouraging physical activity in people with MS can help alleviate symptoms. Similarly, intellectually challenging activities may positively impact cognitive functioning in people with MS.^[1,9]^ Therefore, focusing on entertainment activities in daily adaptive behaviors may be a feasible and economical strategy to improve the overall health of people with MS. While the impact of entertainment activities on other diseases (e.g., Parkinson disease, Alzheimer disease, and cardiovascular disease) has been explored, there is still a lack of epidemiological studies on the causal relationship between recreational activities and MS.^[10–14]^ Given that many entertainment activities are difficult to assess causality through randomized controlled trials, related research is more challenging. Research on the effects of entertainment activities on MS is still in the early stages, and many findings require further validation and in-depth exploration.

When exploring potential causal relationship between entertainment activity factors and the risk of MS, traditional observational epidemiological studies are susceptible to confounding factors, reverse causation and recall bias.^[15]^ To overcome these limitations, Mendelian randomization (MR) provides new perspectives for epidemiological studies. This method strengthens causal inference of risk-outcome associations by utilizing genetic variation as an instrumental variable (IV) for exposure. The basic principle lies in the random assignment of genetic variants during meiosis, making it possible for a trait not to be directly associated with other traits, effectively reducing the influence of confounding factors.^[16,17]^ The two-sample MR analysis enables more precise inference of causality by combining genetic sources of different exposures and outcomes. The statistical efficacy was not only improved, but also confounding bias was significantly reduced. Thus, the MR method and its two-sample application strategy provide a more reliable pathway for causal inference in studies exploring the association between entertainment activity factors and the risk of MS.^[18]^

The objective of this study is to investigate the influence of entertainment activities on MS within individual lifestyles. Here, we conducted a two-sample MR analysis to evaluate the causal impact of 14 entertainment activity factors on the risk of MS. The findings from this study will help not only to improve public awareness of MS risk factors, but also to guide individuals to make healthier choices in their daily lives, thus reducing the incidence of MS.

## 2. Methods

### 2.1. Study design and data sources

Adhering to the comprehensive guidelines of the STROBE-MR checklist,^[19]^ we conducted a two-sample MR analysis. This analysis utilized summary statistics from the publicly available genome-wide association studies (GWAS) on the following 14 entertainment activities. These behaviors encompassed various leisure and social activities, including adult education class (Adult education), pub or social club (Pub/club), religious group (Religious group), sports club or gym (Sports/gym), other group activity (Leisure (other group)), and none of the above (Leisure (None)). Additionally, we analyzed physical activities such as the number of days/week spent walked 10+ minutes (Physical (light)), number of days/week of moderate physical activity 10 + minutes (Physical (moderate)), number of days/week of vigorous physical activity 10 + minutes (Physical (vigorous)). Furthermore, we considered time spent driving (Driving), time spent outdoors in summer (Outdoors (summer)), time spent outdoors in winter (Outdoors (winter)), time spent using computer (Computer), Time spent watching television (TV) (TV); outcome: MS. The study sample sizes varied, ranging from 310,555 to 462,933 individuals, all of whom belonged to the European ancestry. Detailed information on the various traits and the corresponding studies is provided in Table 1. The definitions of all variables are provided in Supplementary Materials (Supplemental Digital Content, https://links.lww.com/MD/Q286).

**Table 1:** Summary of the genome-wide association studies included in this two-sample Mendelian randomization study.

Exposures/ Outcomes	Dataset	Sample size	Number of SNPs	Population	First author/year
Non-cancer illness code, self-reported: multiple sclerosis	ukb-b-17670	462,933	9,851,867	European	Elsworth B/2018
Leisure/social activities: Adult education class	ukb-b-1553	461,369	9,851,867	European	Elsworth B/2018
Leisure/social activities: Pub or social club	ukb-b-4171	461,369	9,851,867	European	Elsworth B/2018
Leisure/social activities: Religious group	ukb-b-4667	461,369	9,851,867	European	Elsworth B/2018
Leisure/social activities: Sports club or gym	ukb-b-4000	461,369	9,851,867	European	Elsworth B/2018
Leisure/social activities: Other group activity	ukb-b-5076	461,369	9,851,867	European	Elsworth B/2018
Leisure/social activities: None of the above	ukb-b-4077	461,369	9,851,867	European	Elsworth B/2018
Time spent doing light physical activity	ukb-b-4886	454,783	9,851,867	European	Elsworth B/2018
Time spent doing moderate physical activity	ukb-b-4710	440,266	9,851,867	European	Elsworth B/2018
Time spent doing vigorous physical activity	ukb-b-151	440,512	9,851,867	European	Elsworth B/2018
Time spent driving	ukb-b-3793	310,555	9,851,867	European	Elsworth B/2018
Time spent outdoors in summer	ukb-b-969	419,314	9,851,867	European	Elsworth B/2018
Time spent outdoors in winter	ukb-b-6811	364,465	9,851,867	European	Elsworth B/2018
Time spent using computer	ukb-b-4522	360,895	9,851,867	European	Elsworth B/2018
Time spent watching television	ukb-b-5192	437,887	9,851,867	European	Elsworth B/2018

### 2.2. IV selection

In this analysis, we selected those that exhibited statistical significance at a stringent *P*-value threshold of less than 5 × 10^−8^. This threshold was based on the published GWAS results for the respective trait, leveraging publicly accessible summary statistics. To guarantee independence among the selected instruments for each trait, we implemented linkage disequilibrium clumping. This process entailed selecting the single nucleotide polymorphism (SNP) with the most significant *P*-value from among all SNPs displaying an linkage disequilibrium *r*^2^ value of 0.001 or greater (Table S1, Supplemental Digital Content, https://links.lww.com/MD/Q286).

### 2.3. Statistical analyses

**Figure 1.**
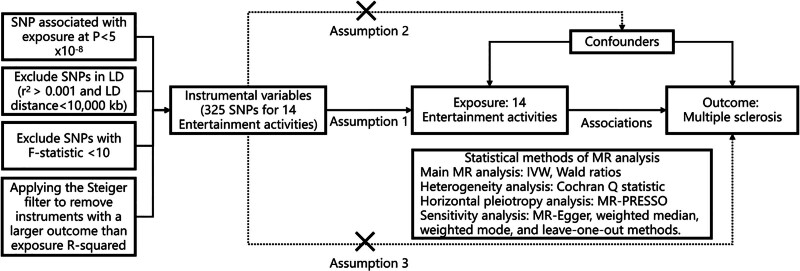
Design and schematic representation of the MR analyses in our study. It shows the detailed steps to study the association between specific genes and MS. With large-scale publicly available GWAS summary statistics, we performed MR analyses to investigate the causal relationship between 14 entertainment activities and MS. To ensure the validity of the IV, 3 assumptions need to be met. Assumption 1: SNPs are significantly correlated with the exposure of interest under study. Assumption 2: SNPs should be independent of any confounding factors that may affect exposure and outcome. Assumption 3: the effect of SNPs on outcome should be captured exclusively through their association with the exposure of interest, with no other independent pathways directly related to the outcome. IV = instrumental variables; IVW = inverse variance weighted, LD = linkage disequilibrium, MR = Mendelian randomization, MR-PRESSO = Mendelian randomization pleiotropy residual sum and outlier, SNP = single nucleotide polymorphism.

For these two-sample MR analyses, we first used the inverse variance weighted (IVW) random effects approach. This approach served as a meta-analysis, combining the variant-specific Wald ratios for each SNP to estimate the overall causal effect. While the IVW approach assumes independence and validity of IVs, it may ignore mediating effects or potential pleiotropy arising from other risk factors. In cases where horizontal pleiotropy is present among the instrumental SNPs, this can introduce bias and violate the IV assumptions.^[20,21]^ Therefore, we additionally applied MR-Egger, weighted median, and weighted mode methods to assess robustness.^[19]^ The MR-Egger method assumes that there is no correlation between the magnitude of pleiotropic effects and the strength of genetic-phenotype associations across all instruments.^[21]^ Weighted modal method relies on a subset of valid variables that exhibit consistent causal effects, and the weighted median method assigns 50% weight to variables from valid instruments.^[22]^ These supplementary analyses aim to assess the robustness of our findings, taking into account the potential presence of pleiotropic effects. We tested for heterogeneity using Cochran *Q* statistic, with significance set at *P* < .05. Furthermore, we assessed horizontal pleiotropy using the MR-PRESSO analysis where *P* < .05 indicates its presence. All statistical analyses were performed using R 3.6.0 with the TwoSampleMR package (https://github.com/MRCIEU/TwoSampleMR; RRID: SCR_019010) (Fig. 1).

## 3. Results

**Figure 2.**
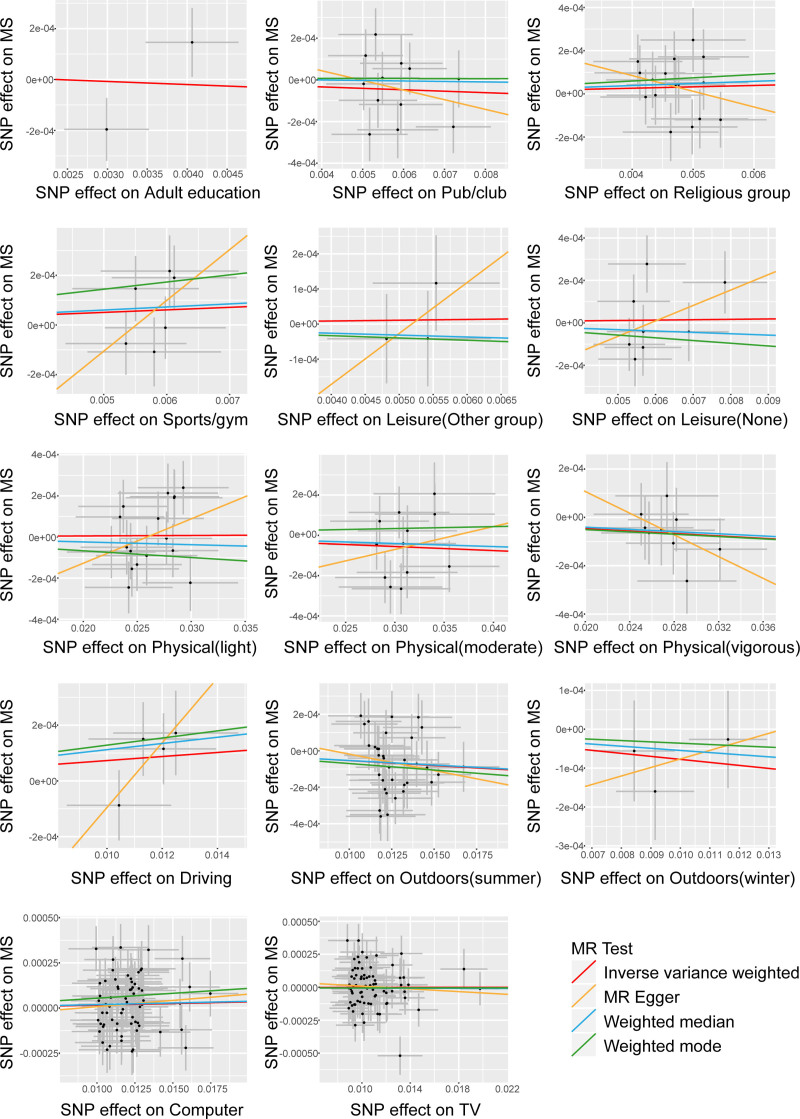
The effect of entertainment activities on MS. Results are from the IVW method of the two-sample MR analysis. * Shows statistically significant correlation with causal effect. The size of the box represents the weight of the study. CI = confidence interval; *H*-pval = heterogeneity *P*-value, OR = odds ratios, *P*-pval = horizontal pleiotropy *P*-value or pleiotropy *P*-value, S-pval = Steiger test *P*-value, SNP = single nucleotide polymorphism.

**Figure 3.**
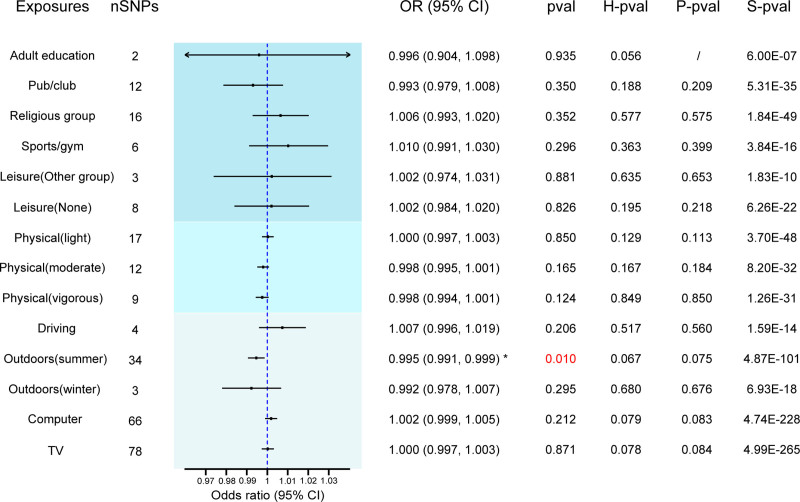
Assessment of significant causality in genetic associations between entertainment activities factors and MS. Each data point illustrates the relevant change at the trait level and the increased risk for each genetic variant. Horizontal coordinates indicate the number of genetic variants associated with exposure factors and vertical coordinates indicate the number of genetic variants associated with disease risk. The horizontal and vertical lines for each point indicate the 95% CI of the corresponding genetic association. The colored lines indicate the trait slopes of the results obtained by applying various different MR methods. MR = Mendelian randomization, MS = multiple sclerosis, SNP = single nucleotide polymorphism.

The *F*-statistics for all IVs exceeded 10, indicating the absence of weak instrument bias in our analysis (Table S1, Supplemental Digital Content, https://links.lww.com/MD/Q286). Utilizing the IVW random effects method, we analyzed the associations between 14 entertainment activities and MS. Figure 2 shows the forest plot illustrating the association between the risk of MS and each entertainment activity. In addition, Figure 3 displays scatterplots of the associations of genetic variants with their corresponding outcomes. Our analysis revealed significant causal effect of time spent outdoors in summer on MS (OR = 0.995; 95% CI = 0.991–0.999; *P* = .010). Notably, no significant correlations were found between MS and leisure/social activities, weekly exercise, time spent driving, time spent outdoors in winter, time spent using computer or watching television with MS. The full results of our study are available in Table S2 (Supplemental Digital Content, https://links.lww.com/MD/Q286). The leave–one–out sensitivity further corroborated the aforementioned conclusion (Table S3, Supplemental Digital Content, https://links.lww.com/MD/Q286). We didn’t observe Cochrane *Q* statistic in the sensitivity test (*P* = .067), which indicates the robustness of the data (Table S4, Supplemental Digital Content, https://links.lww.com/MD/Q286). In the Mendelian randomization pleiotropy residual sum and outlier (MR-PRESSO) analysis (*P* = .075), there was no presence of horizontal pleiotropy effect, supporting the reliability of the data (Table S5, Supplemental Digital Content, https://links.lww.com/MD/Q286).

## 4. Discussion

In conclusion, our two-sample MR study revealed a notable association between genetically predicted time spent outdoors in summer and the risk of MS. Our results indicate that time spent outdoors in summer is associated with a reduced risk of MS. Nonetheless, our analysis failed to find evidence to support the supposition that leisure or social activities, weekly exercise, time spent driving, time outdoors in winter, time spent using computer or watching television have a direct causal link with the risk of developing MS.

Previous studies have provided insights into the association between time spent outdoors in summer and MS. Evidence shows that inadequate ultraviolet exposure is a modifiable risk factor for MS. Ultraviolet light exposure promotes the synthesis of vitamin D in the skin, which is widely regarded as a protective factor for MS. Specifically, higher vitamin D levels and ultraviolet exposure correlate with a reduced risk of MS.^[5,23,24]^ Ultraviolet exposure reduces peripheral inflammation in mice, enhances T regulatory cell activation, and may promote cis-urocanic acid production by affecting dendritic cells. Therefore, moderate sunlight exposure is recommended. It is necessary to increase sun exposure or supplement vitamin D during the winter months when sunlight is less available.^[25–27]^ Additionally exercise may play a protective role by influencing the regulation of immune factors and stress hormones in MS.^[28]^ Appropriate exercise not only improves blood circulation, muscle strength and endurance, but also improves cognitive function and enhances cardiovascular function.^[29–31]^ From both prevention and treatment perspectives, outdoor activities should be promoted among people at high risk of MS and patients with MS. More outdoor activities can positively influence the progression of MS and significantly improve quality of life.^[6]^ But when in outdoors, take care to avoid exposure to the sun and long periods of exercise to reduce the risk of ultraviolet damage to the skin and eyes. MS is a chronic inflammatory demyelinating disease of the central nervous system. For patients prone to relapse, reducing relapse frequency and delaying disease progression constitute key therapeutic objectives. Endogenous remyelination promotion has emerged as one of the most promising therapeutic approaches for MS, given its potential to restore neuronal function and prevent further neuronal loss.^[32]^ Future research should further investigate the specific mechanisms underlying outdoor activity’s effects in relapse-prone patients. For instance, clinical trials could examine whether increased outdoor exposure reduces relapse frequency or improves neurological dysfunction in this population. Additionally, research should also explore the potential synergistic effects between outdoor activities and other therapeutic interventions, such as pharmacological treatments and rehabilitation training, to optimize treatment outcomes. The Sigma-1 receptor plays a critical role in endoplasmic reticulum (ER) stress response by modulating ER-mitochondrial function, thereby protecting cells from stress-induced damage. Outdoor activity may indirectly activate Sigma-1 receptors through neuroendocrine system modulation, alleviating ER stress. Sigma-1 receptor activation reduces oxidative stress and apoptosis, conferring neuroprotection. Enhanced physical activity and improved circulation through outdoor exposure may elevate neuronal antioxidant capacity, potentiating the receptor’s neuroprotective effects.^[33]^ Furthermore, Sigma-1 receptors contribute significantly to neuroplasticity by regulating synaptic stability and function. Outdoor activity may promote neuroplasticity through increased cerebral neural activity, thereby augmenting Sigma-1 receptor functionality.^[34,35]^

In discussing the relationship between MS and level of physical activity, our findings provide corroboration of previous research. There was no causal relationship between too light or too vigorous exercise and MS.^[36]^ Our results also present some discrepancies with previous conclusions. Although previous studies have revealed that leisure/social activities have a positive impact on people with MS and that there is some correlation between cognitive abilities and participation in social and intellectual activities in people with MS. However, we did not observe such significant correlation in this study.^[37]^ The findings are inconsistent possibly because the susceptibility of MS to lifestyle factors may be evident years before the clinical episode, complicating epidemiological studies. Notably, these results are not universally applicable to all MS patients, especially considering different MS types, expanded disability status scale scores, and individual differences. Moreover, the lack of long-term follow-up data makes it difficult to fully assess the lasting effects of our interventions.

The study possesses both strengths and limitations. It covers a comprehensive range of entertainment activity factors and boasts a substantial sample size, representing a significant strength. In terms of data processing, we evaluated summary data and failed to explore individual-specific data in depth, which limited our assessment of specific genetic variation at the individual level. The relatively small number of genetic instruments (<10 SNPs each) for some of the traits in this study increases the risk of weak instrumentation bias, which may have some impact on the precision and reliability of the study. In addition, the population of the study focused on individuals of European ancestry, and genetic and prevalence differences between races may limit the applicability of our findings to other racial groups.^[38]^ All in all, entertainment activities in daily adaptive behaviors may become a feasible strategy to improve the overall health of people with MS. Future studies can further explore the association between entertainment activities and MS to find out more effective prevention strategies. In the meantime, policy makers and health promotion organizations can promote healthy entertainment activities and lifestyles to improve public health and reduce the risk of MS.

## 5. Conclusion

In summary, our findings reveal a potential causal relationship between time spent outdoors in summer and MS, offering crucial implications for preventive measures against this disease. This result may serve as a catalyst for the formulation of targeted prevention strategies. However, further interventional studies are essential to gain a deeper understanding of the underlying mechanisms.

## Acknowledgments

The authors thank the studies or consortiums cited and included in this analysis for providing public datasets. The authors are also grateful to all those who participated in this study and wish to acknowledge the valuable assistance obtained from all specialized researchers.

## Author contributions

**Conceptualization:** Zheng Wang.

**Data curation:** Zheng Wang.

**Formal analysis:** Zheng Wang.

**Investigation:** Zheng Wang.

**Methodology:** Zheng Wang.

**Project administration:** Wan Wu.

**Resources:** Zheng Wang.

**Software:** Zheng Wang.

**Supervision:** Wan Wu.

**Validation:** Wan Wu.

**Visualization:** Zheng Wang.

**Writing – original draft:** Zheng Wang.

**Writing – review & editing:** Zheng Wang.

## Supplementary Material


