# Supplementary material for: Entertainment activities and the risk of multiple sclerosis: A Mendelian randomization analysis
Source: Medicine (Baltimore). 2025 Oct 17;104(42):e44981. doi: 10.1097/MD.0000000000044981 (PMC12537092; doi:10.1097/MD.0000000000044981)
Supplement: Supplementary file 1 [file medi-104-e44981-s001.pdf]

Supplementary Table 1: The F-statistics for all instrumental variables in this two-sample mendelian randomization study.

SNP	id.outcome	pval.outcome	id.exposure	steiger_pval	R^2	F-statistics
rs1491872	ukb-b-17670	0.74	ukb-b-151	8.52E-05	0.000291717	33.72454305
rs2005617	ukb-b-17670	0.92	ukb-b-151	3.66E-05	0.00029355	34.38040949
rs2189464	ukb-b-17670	0.719999	ukb-b-151	0.000189425	0.000268549	31.03094995
rs2764261	ukb-b-17670	0.41	ukb-b-151	4.28E-05	0.000365158	42.55238759
rs328900	ukb-b-17670	0.051	ukb-b-151	0.000924716	0.000366324	42.68695197
rs382210	ukb-b-17670	0.94	ukb-b-151	4.52E-06	0.000359488	41.93862668
rs6533635	ukb-b-17670	0.62	ukb-b-151	8.06E-05	0.000310723	35.89105818
rs6955240	ukb-b-17670	0.3	ukb-b-151	2.66E-06	0.000490755	57.17936483
rs7072776	ukb-b-17670	0.53	ukb-b-151	0.000155238	0.00030048	34.85410716
rs1125000	ukb-b-17670	0.11	ukb-b-1553	0.004212087	4.47E-06	31.63656551
rs6997	ukb-b-17670	0.28	ukb-b-1553	2.93E-05	7.01E-06	48.72784927
rs11057408	ukb-b-17670	0.26	ukb-b-3793	0.000222031	5.69E-05	32.52961576
rs12069474	ukb-b-17670	0.26	ukb-b-3793	0.000392287	5.35E-05	30.38093022
rs12921753	ukb-b-17670	0.37	ukb-b-3793	1.66E-05	6.96E-05	39.76134557
rs2588917	ukb-b-17670	0.49	ukb-b-3793	0.000127788	5.39E-05	30.47705765
rs11712056	ukb-b-17670	0.93	ukb-b-4000	1.41E-05	1.76E-05	38.73280694
rs13002862	ukb-b-17670	0.13	ukb-b-4000	0.004578215	1.38E-05	30.4578794
rs2499760	ukb-b-17670	0.55	ukb-b-4000	0.000426875	1.43E-05	31.03762821
rs62263912	ukb-b-17670	0.14	ukb-b-4000	0.000870712	1.75E-05	38.02457097
rs6478444	ukb-b-17670	0.27	ukb-b-4000	0.001978087	1.38E-05	30.07081052
rs7228990	ukb-b-17670	0.44	ukb-b-4000	0.000899962	1.37E-05	29.92059254
rs11917431	ukb-b-17670	0.39	ukb-b-4077	0.000323943	1.29E-05	35.2093695
rs17824247	ukb-b-17670	0.74	ukb-b-4077	0.000242218	1.12E-05	30.40781695
rs2773485	ukb-b-17670	0.760001	ukb-b-4077	9.66E-05	1.25E-05	33.78950925
rs10978543	ukb-b-17670	0.57	ukb-b-4171	0.00016921	1.42E-05	34.62879682

				5		
rs12103006	ukb-b-17670	0.88	ukb-b-4171	0.00016072<br>1	1.24E-05	30.07280603
rs12472555	ukb-b-17670	0.94	ukb-b-4171	2.56E-05	1.50E-05	36.29341631
rs12759477	ukb-b-17670	0.97	ukb-b-4171	1.84E-07	2.26E-05	54.80326589
rs139920	ukb-b-17670	0.67	ukb-b-4171	7.14E-06	1.89E-05	45.91263375
rs2661863	ukb-b-17670	0.46	ukb-b-4171	0.00056116<br>8	1.29E-05	31.54801802
rs36030660	ukb-b-17670	0.4	ukb-b-4171	0.00044760<br>1	1.38E-05	33.58898901
rs61873510	ukb-b-17670	0.079000<br>5	ukb-b-4171	0.00316554<br>2	1.51E-05	35.07743385
rs6969458	ukb-b-17670	0.074999<br>8	ukb-b-4171	1.63E-05	2.59E-05	61.93800555
rs754204	ukb-b-17670	0.36	ukb-b-4171	0.00107956<br>8	1.29E-05	30.71892421
rs780094	ukb-b-17670	0.042000<br>1	ukb-b-4171	0.01260209	1.27E-05	30.89134672
rs9401593	ukb-b-17670	0.081999<br>3	ukb-b-4171	0.00368030<br>2	1.41E-05	34.07563999
rs10208088	ukb-b-17670	0.49	ukb-b-4522	0.00021764<br>6	5.11E-05	30.68536895
rs1037091	ukb-b-17670	0.85	ukb-b-4522	1.02E-08	0.000105492	60.91811788
rs10518019	ukb-b-17670	0.008700<br>01	ukb-b-4522	0.01874277	4.93E-05	29.73799189
rs10828248	ukb-b-17670	0.84	ukb-b-4522	5.59E-05	5.13E-05	30.84630653
rs11634155	ukb-b-17670	0.17	ukb-b-4522	0.00035587<br>7	6.01E-05	35.55578882
rs11652437	ukb-b-17670	0.47	ukb-b-4522	1.28E-05	7.15E-05	41.77254068
rs117405403	ukb-b-17670	0.22	ukb-b-4522	9.84E-05	6.56E-05	39.55703682
rs11749912	ukb-b-17670	0.099001<br>1	ukb-b-4522	3.85E-05	8.11E-05	48.25616479
rs11766392	ukb-b-17670	0.15	ukb-b-4522	0.00016271<br>2	6.61E-05	39.58461878
rs11942953	ukb-b-17670	0.47	ukb-b-4522	0.00015792<br>1	5.44E-05	32.32400634
rs12128707	ukb-b-17670	0.21	ukb-b-4522	0.00046241<br>6	5.63E-05	33.51753652
rs12145677	ukb-b-17670	0.38	ukb-b-4522	1.43E-07	0.000101388	60.7621385
rs12521638	ukb-b-17670	0.59	ukb-b-4522	0.00017236<br>9	5.02E-05	30.05578954
rs12553324	ukb-b-17670	0.53	ukb-b-4522	2.85E-11	0.000148363	89.02638377
rs12706626	ukb-b-17670	0.89	ukb-b-4522	4.71E-05	5.12E-05	30.76179609

rs12820967	ukb-b-17670	0.7	ukb-b-4522	1.93E-05	6.18E-05	36.55661385
rs12946454	ukb-b-17670	0.25	ukb-b-4522	0.000229489	5.88E-05	35.12350782
rs13262595	ukb-b-17670	0.0299999	ukb-b-4522	7.81E-07	0.00012038	72.3706469
rs136553	ukb-b-17670	0.35	ukb-b-4522	0.000107745	6.01E-05	35.99414431
rs1395020	ukb-b-17670	0.630001	ukb-b-4522	0.000132546	5.11E-05	30.46565799
rs1448355	ukb-b-17670	0.5	ukb-b-4522	9.42E-06	7.09E-05	42.26268361
rs1469249	ukb-b-17670	0.9	ukb-b-4522	2.22E-05	5.60E-05	33.27669978
rs1648906	ukb-b-17670	0.12	ukb-b-4522	0.001508405	5.32E-05	31.42830369
rs166835	ukb-b-17670	0.95	ukb-b-4522	1.32E-05	5.78E-05	34.48101711
rs17167210	ukb-b-17670	0.39	ukb-b-4522	4.57E-05	6.39E-05	38.30653336
rs17789218	ukb-b-17670	0.57	ukb-b-4522	3.85E-05	5.97E-05	35.8717117
rs17862355	ukb-b-17670	0.21	ukb-b-4522	0.000182691	6.20E-05	37.24284236
rs1987942	ukb-b-17670	0.0389996	ukb-b-4522	0.002632853	5.78E-05	34.0466585
rs1999244	ukb-b-17670	0.780001	ukb-b-4522	2.07E-05	5.87E-05	35.06505079
rs2032780	ukb-b-17670	0.38	ukb-b-4522	1.36E-05	7.36E-05	43.37398173
rs2041687	ukb-b-17670	0.96	ukb-b-4522	3.18E-06	6.79E-05	39.23042302
rs2068625	ukb-b-17670	0.33	ukb-b-4522	2.65E-06	8.46E-05	50.77581554
rs206965	ukb-b-17670	0.16	ukb-b-4522	0.000700976	5.54E-05	33.29666623
rs2120461	ukb-b-17670	0.450001	ukb-b-4522	2.28E-05	6.65E-05	40.00260777
rs2220599	ukb-b-17670	0.0649995	ukb-b-4522	0.000266879	7.06E-05	42.14030491
rs246723	ukb-b-17670	0.0719996	ukb-b-4522	0.002048329	5.50E-05	32.50236367
rs2588543	ukb-b-17670	0.66	ukb-b-4522	9.48E-05	5.25E-05	31.2867388
rs2734833	ukb-b-17670	0.0719996	ukb-b-4522	0.0001651	7.31E-05	43.72566769
rs2748985	ukb-b-17670	0.29	ukb-b-4522	1.04E-05	7.75E-05	46.44721799
rs2756121	ukb-b-17670	0.01	ukb-b-4522	0.004309443	6.21E-05	37.00187704
rs2761438	ukb-b-17670	0.5	ukb-b-4522	4.68E-05	6.03E-05	36.27280737
rs28710456	ukb-b-17670	0.41	ukb-b-4522	0.000195599	5.41E-05	32.42607429
rs306755	ukb-b-17670	0.13	ukb-b-4522	0.001186808	5.37E-05	32.17852154
rs422115	ukb-b-17670	0.48	ukb-b-4522	0.00010432	5.65E-05	33.69808656

				4		
rs4704043	ukb-b-17670	0.84	ukb-b-4522	2.92E-05	5.51E-05	33.07519239
rs4852252	ukb-b-17670	0.28	ukb-b-4522	0.00059541 2	5.11E-05	30.71842941
rs56229818	ukb-b-17670	0.75	ukb-b-4522	8.13E-05	5.12E-05	30.71542151
rs58638214	ukb-b-17670	0.96	ukb-b-4522	3.20E-07	7.89E-05	47.11738545
rs6028090	ukb-b-17670	0.55	ukb-b-4522	2.89E-06	7.76E-05	45.87002768
rs6449708	ukb-b-17670	0.87	ukb-b-4522	2.50E-05	5.57E-05	33.302234
rs6780848	ukb-b-17670	0.4	ukb-b-4522	0.00032982 6	5.09E-05	30.54166507
rs6935828	ukb-b-17670	0.3	ukb-b-4522	0.00052856 2	5.10E-05	30.64997882
rs7020477	ukb-b-17670	0.82	ukb-b-4522	2.80E-05	5.61E-05	33.54796516
rs7209653	ukb-b-17670	0.018999 8	ukb-b-4522	0.00054088 5	7.46E-05	44.77184748
rs7281293	ukb-b-17670	0.39	ukb-b-4522	0.00010458 8	5.94E-05	35.18930823
rs7288455	ukb-b-17670	0.23	ukb-b-4522	0.00047110 8	5.46E-05	32.64687089
rs73578186	ukb-b-17670	0.28	ukb-b-4522	6.69E-05	6.62E-05	39.26452607
rs7526112	ukb-b-17670	0.1	ukb-b-4522	0.00132944 3	5.43E-05	32.65323323
rs7564844	ukb-b-17670	0.709999	ukb-b-4522	1.07E-05	6.39E-05	38.45278393
rs7630869	ukb-b-17670	0.39	ukb-b-4522	3.15E-08	0.000109919	66.24910228
rs7904398	ukb-b-17670	0.59	ukb-b-4522	0.00012907 7	5.20E-05	31.1355453
rs7968738	ukb-b-17670	0.58	ukb-b-4522	4.02E-05	5.99E-05	35.65886328
rs79720045	ukb-b-17670	0.41	ukb-b-4522	7.11E-06	7.82E-05	45.23061312
rs806795	ukb-b-17670	0.83	ukb-b-4522	3.83E-05	5.34E-05	32.23026724
rs8102851	ukb-b-17670	0.53	ukb-b-4522	9.17E-05	5.72E-05	33.38296777
rs9375188	ukb-b-17670	0.079000 5	ukb-b-4522	1.01E-07	0.000125509	74.89626732
rs12156017	ukb-b-17670	0.74	ukb-b-4667	3.26E-05	1.03E-05	38.40583704
rs12992090	ukb-b-17670	0.23	ukb-b-4667	0.00010355 4	1.19E-05	44.72508843
rs1471093	ukb-b-17670	0.35	ukb-b-4667	8.59E-06	1.41E-05	52.07737835
rs17527878	ukb-b-17670	0.47	ukb-b-4667	0.00021642 5	9.49E-06	35.3643057
rs1991083	ukb-b-17670	0.19	ukb-b-4667	0.00121353 5	9.34E-06	34.62189511
rs3197999	ukb-b-17670	0.760001	ukb-b-4667	9.28E-05	9.09E-06	33.97343465
rs332828	ukb-b-17670	0.68	ukb-b-4667	2.62E-06	1.33E-05	49.68330012
rs36104984	ukb-b-17670	0.62	ukb-b-4667	0.00024111	8.67E-06	32.28410057

				2		
rs410671	ukb-b-17670	0.18	ukb-b-4667	7.86E-05	1.30E-05	47.83489724
rs6545977	ukb-b-17670	0.23	ukb-b-4667	0.001840269	8.37E-06	31.317246
rs6722794	ukb-b-17670	0.91	ukb-b-4667	8.38E-05	8.66E-06	32.18964524
rs6862251	ukb-b-17670	0.39	ukb-b-4667	8.46E-05	1.10E-05	41.03211365
rs699534	ukb-b-17670	0.2	ukb-b-4667	0.000357565	1.08E-05	39.87959593
rs7388625	ukb-b-17670	0.450001	ukb-b-4667	0.00075255	8.16E-06	30.47560297
rs8020432	ukb-b-17670	0.96	ukb-b-4667	0.000127691	8.08E-06	29.80658298
rs990702	ukb-b-17670	0.0909997	ukb-b-4667	0.003628197	9.11E-06	33.61412647
rs10098073	ukb-b-17670	0.0350002	ukb-b-4710	0.00365602	0.000467707	37.40298346
rs1036800	ukb-b-17670	0.18	ukb-b-4710	0.002585638	0.000384903	30.28060625
rs11749912	ukb-b-17670	0.0990011	ukb-b-4710	0.003088477	0.000410998	32.95324588
rs2246122	ukb-b-17670	0.58	ukb-b-4710	0.000239309	0.000400623	32.1800765
rs404907	ukb-b-17670	0.86	ukb-b-4710	1.47E-05	0.000485925	38.77553019
rs4129572	ukb-b-17670	0.23	ukb-b-4710	3.22E-05	0.000607514	48.81701037
rs4540651	ukb-b-17670	0.719999	ukb-b-4710	0.000145175	0.000395928	32.02045687
rs4886868	ukb-b-17670	0.38	ukb-b-4710	0.000267945	0.000449512	35.4456631
rs7229874	ukb-b-17670	0.49	ukb-b-4710	0.000416679	0.000395496	31.34223158
rs7565480	ukb-b-17670	0.0519996	ukb-b-4710	0.007515553	0.000391471	31.67777012
rs9533455	ukb-b-17670	0.760001	ukb-b-4710	9.23E-05	0.000408782	33.16567396
rs997467	ukb-b-17670	0.15	ukb-b-4710	0.00072832	0.000478135	37.41727671
rs11183184	ukb-b-17670	0.44	ukb-b-4886	0.000452095	0.000272575	32.47366697
rs12568280	ukb-b-17670	0.3	ukb-b-4886	0.000406055	0.000297078	36.18829202
rs12979056	ukb-b-17670	0.051	ukb-b-4886	0.004349322	0.000290877	35.4205741
rs1455343	ukb-b-17670	0.61	ukb-b-4886	1.01E-05	0.000377136	45.18279561
rs2011071	ukb-b-17670	0.25	ukb-b-4886	0.001262749	0.000268603	32.27205202
rs2220599	ukb-b-17670	0.064999	ukb-b-4886	0.00026817	0.000398564	48.42781177

		5		8		
rs34654885	ukb-b-17670	0.5	ukb-b-4886	5.86E-05	0.000326748	40.11503258
rs4588066	ukb-b-17670	0.25	ukb-b-4886	0.00149838 6	0.000257885	31.46591783
rs4641022	ukb-b-17670	0.709999	ukb-b-4886	0.00019937 3	0.000257256	31.49405238
rs62172117	ukb-b-17670	0.96	ukb-b-4886	3.79E-06	0.000352707	43.03066043
rs62422661	ukb-b-17670	0.13	ukb-b-4886	0.00014411 6	0.000386236	47.20361796
rs62482241	ukb-b-17670	0.14	ukb-b-4886	0.00132217 9	0.000294811	35.94260032
rs627685	ukb-b-17670	0.1	ukb-b-4886	0.00026009 5	0.000378451	45.62908683
rs72931435	ukb-b-17670	0.53	ukb-b-4886	0.00051219 1	0.000248246	30.38977117
rs747344	ukb-b-17670	0.57	ukb-b-4886	0.00045988 4	0.000248121	30.27856161
rs7576657	ukb-b-17670	0.17	ukb-b-4886	0.00040182 1	0.000336101	40.32214392
rs9400239	ukb-b-17670	0.62	ukb-b-4886	0.00038117 8	0.000247342	30.25344937
rs13011181	ukb-b-17670	0.19	ukb-b-5076	5.68E-05	2.24E-05	48.77477061
rs2043145	ukb-b-17670	0.75	ukb-b-5076	8.95E-05	1.57E-05	34.34017574
rs2675638	ukb-b-17670	0.42	ukb-b-5076	0.00067172 9	1.43E-05	31.42053107
rs3197999	ukb-b-17670	0.760001	ukb-b-5076	1.15E-05	1.94E-05	42.30018068
rs35291206	ukb-b-17670	0.39	ukb-b-5076	0.00093947 5	1.43E-05	30.67306518
rs410671	ukb-b-17670	0.18	ukb-b-5076	0.00271096 8	1.44E-05	31.06956998
rs4247450	ukb-b-17670	0.04	ukb-b-5076	0.01242709	1.43E-05	31.18447584
rs847687	ukb-b-17670	0.42	ukb-b-5076	0.00081394 7	1.40E-05	30.69956031
rs10109061	ukb-b-17670	0.88	ukb-b-5192	0.00011014 3	3.97E-05	30.67227791
rs10189857	ukb-b-17670	0.17	ukb-b-5192	2.00E-08	0.000105867	83.71824169
rs10269099	ukb-b-17670	0.53	ukb-b-5192	0.00034594 3	3.99E-05	31.41190546
rs10739499	ukb-b-17670	0.33	ukb-b-5192	0.00116855 3	3.81E-05	30.04955035
rs10765777	ukb-b-17670	0.12	ukb-b-5192	0.00011564 2	6.09E-05	47.73019348
rs11191129	ukb-b-17670	0.38	ukb-b-5192	0.00015952	4.73E-05	37.4663044

				5		
rs11245482	ukb-b-17670	0.48	ukb-b-5192	7.30E-05	4.92E-05	38.77207899
rs11662211	ukb-b-17670	0.021	ukb-b-5192	0.008004831	4.46E-05	35.2425516
rs11680095	ukb-b-17670	0.28	ukb-b-5192	0.00103573	4.08E-05	31.61915977
rs11700249	ukb-b-17670	0.3	ukb-b-5192	0.00056332	4.30E-05	33.86022601
rs11714337	ukb-b-17670	0.61	ukb-b-5192	4.98E-05	4.82E-05	37.85503452
rs11877758	ukb-b-17670	0.61	ukb-b-5192	2.10E-05	5.27E-05	41.28487465
rs11911112	ukb-b-17670	0.33	ukb-b-5192	7.00E-05	5.36E-05	42.24760103
rs12214364	ukb-b-17670	0.38	ukb-b-5192	0.000881805	3.97E-05	30.23402171
rs12553324	ukb-b-17670	0.53	ukb-b-5192	2.42E-08	8.94E-05	70.52360166
rs1291871	ukb-b-17670	0.8	ukb-b-5192	8.28E-05	4.19E-05	32.86168483
rs13014947	ukb-b-17670	0.53	ukb-b-5192	2.24E-05	5.50E-05	42.66294777
rs1727332	ukb-b-17670	0.000350002	ukb-b-5192	0.009151149	6.42E-05	50.60122673
rs17789218	ukb-b-17670	0.57	ukb-b-5192	0.000123877	4.40E-05	34.84526376
rs178203	ukb-b-17670	0.98	ukb-b-5192	3.01E-06	5.42E-05	42.84494635
rs180396	ukb-b-17670	0.16	ukb-b-5192	0.003175404	3.82E-05	30.00742679
rs1993092	ukb-b-17670	0.91	ukb-b-5192	4.07E-05	4.31E-05	34.05139909
rs2106164	ukb-b-17670	0.3	ukb-b-5192	0.000486129	4.39E-05	34.51327245
rs2185490	ukb-b-17670	0.61	ukb-b-5192	0.000193281	4.15E-05	32.47080415
rs2283	ukb-b-17670	0.16	ukb-b-5192	0.002761244	3.85E-05	30.59186767
rs2332818	ukb-b-17670	0.77	ukb-b-5192	0.000187078	3.84E-05	30.17223312
rs2352984	ukb-b-17670	0.93	ukb-b-5192	1.80E-18	0.000191449	151.7030625
rs262890	ukb-b-17670	0.9	ukb-b-5192	7.69E-08	7.35E-05	58.04220372
rs263771	ukb-b-17670	0.780001	ukb-b-5192	1.25E-05	5.17E-05	40.60191896
rs2646351	ukb-b-17670	0.21	ukb-b-5192	0.001833658	3.94E-05	31.10148592
rs2678662	ukb-b-17670	0.64	ukb-b-5192	4.93E-06	5.92E-05	46.62082574
rs2725371	ukb-b-17670	0.061	ukb-b-5192	2.94E-05	7.45E-05	58.478909
rs2906604	ukb-b-17670	0.56	ukb-b-5192	1.73E-05	5.41E-05	42.99393518
rs3138499	ukb-b-17670	0.86	ukb-b-5192	1.80E-06	6.00E-05	46.65829264
rs34094119	ukb-b-17670	0.91	ukb-b-5192	6.21E-05	4.11E-05	32.38190809
rs34811474	ukb-b-17670	0.630001	ukb-b-5192	3.12E-06	6.14E-05	48.5984564
rs35797019	ukb-b-17670	0.719999	ukb-b-5192	0.000138537	4.05E-05	32.03995188

rs362312	ukb-b-17670	0.47	ukb-b-5192	0.000190059	4.43E-05	34.89259236
rs3754970	ukb-b-17670	0.99	ukb-b-5192	1.41E-05	4.71E-05	36.82830538
rs3810496	ukb-b-17670	0.0980009	ukb-b-5192	0.003381377	4.15E-05	32.48263735
rs4076457	ukb-b-17670	0.0619998	ukb-b-5192	0.008126499	3.85E-05	30.29860774
rs4110177	ukb-b-17670	0.27	ukb-b-5192	0.000951297	4.13E-05	32.37491381
rs4303732	ukb-b-17670	0.24	ukb-b-5192	8.84E-05	5.53E-05	43.77946022
rs4339469	ukb-b-17670	0.19	ukb-b-5192	5.50E-06	7.32E-05	57.8607811
rs4469687	ukb-b-17670	0.00460002	ukb-b-5192	0.046514516	3.87E-05	30.62089589
rs4747438	ukb-b-17670	0.82	ukb-b-5192	1.45E-06	6.11E-05	48.15249195
rs4788616	ukb-b-17670	0.35	ukb-b-5192	0.000129317	4.94E-05	38.98178372
rs4847408	ukb-b-17670	0.0840001	ukb-b-5192	0.001051728	4.92E-05	39.06813679
rs494566	ukb-b-17670	0.630001	ukb-b-5192	7.54E-05	4.58E-05	35.92984222
rs57555420	ukb-b-17670	0.0539995	ukb-b-5192	0.005536796	4.19E-05	32.95692001
rs6102912	ukb-b-17670	0.89	ukb-b-5192	2.11E-06	5.76E-05	45.6027693
rs61864793	ukb-b-17670	0.86	ukb-b-5192	2.31E-05	4.68E-05	36.89428755
rs62145951	ukb-b-17670	0.38	ukb-b-5192	3.14E-05	5.61E-05	44.43286618
rs62199883	ukb-b-17670	0.88	ukb-b-5192	8.48E-10	9.57E-05	75.76840498
rs6511708	ukb-b-17670	0.36	ukb-b-5192	6.65E-06	6.51E-05	51.41040117
rs6814554	ukb-b-17670	0.79	ukb-b-5192	3.91E-09	9.13E-05	71.82265439
rs6850494	ukb-b-17670	0.27	ukb-b-5192	0.000538445	4.42E-05	34.93805335
rs6994132	ukb-b-17670	0.53	ukb-b-5192	6.52E-05	4.84E-05	38.25000022
rs7089973	ukb-b-17670	1	ukb-b-5192	5.25E-05	4.07E-05	31.89182451
rs7184800	ukb-b-17670	0.59	ukb-b-5192	2.04E-07	7.64E-05	60.4760458
rs749056	ukb-b-17670	0.29	ukb-b-5192	0.000631175	4.25E-05	33.48162593
rs749671	ukb-b-17670	0.69	ukb-b-5192	5.35E-06	5.74E-05	45.38782672
rs7539775	ukb-b-17670	0.8	ukb-b-5192	0.000177938	3.79E-05	30.00513103
rs75499503	ukb-b-17670	0.37	ukb-b-5192	7.71E-10	0.000116432	89.46347576
rs7708324	ukb-b-17670	0.99	ukb-b-5192	1.60E-05	4.59E-05	36.40410074
rs7798292	ukb-b-17670	0.005	ukb-b-5192	0.016961207	4.64E-05	36.76416767
rs7899206	ukb-b-17670	0.13	ukb-b-5192	0.00094042	4.78E-05	36.85235109
rs7921305	ukb-b-17670	0.16	ukb-b-5192	0.00046314	4.97E-05	39.25694888

				1		
rs801733	ukb-b-17670	0.82	ukb-b-5192	1.61E-07	7.17E-05	56.7232424
rs814197	ukb-b-17670	0.91	ukb-b-5192	2.24E-06	5.68E-05	44.94816636
rs883027	ukb-b-17670	0.0700003	ukb-b-5192	0.002390162	4.56E-05	36.01629659
rs898751	ukb-b-17670	0.56	ukb-b-5192	3.93E-05	5.02E-05	39.66448277
rs9300594	ukb-b-17670	0.0940005	ukb-b-5192	0.001274438	4.75E-05	37.5045759
rs9471333	ukb-b-17670	0.95	ukb-b-5192	3.60E-06	5.38E-05	42.59205823
rs9834970	ukb-b-17670	0.96	ukb-b-5192	6.59E-05	3.97E-05	31.49091496
rs9867437	ukb-b-17670	0.94	ukb-b-5192	5.23E-06	5.28E-05	41.36609177
rs9880023	ukb-b-17670	0.41	ukb-b-5192	0.000194769	4.59E-05	35.99395533
rs996234	ukb-b-17670	0.34	ukb-b-5192	8.81E-05	5.55E-05	40.91592883
rs12956276	ukb-b-17670	0.67	ukb-b-6811	1.63E-05	3.26E-05	37.77121139
rs1368549	ukb-b-17670	0.2	ukb-b-6811	1.59E-05	4.18E-05	47.67753951
rs4580876	ukb-b-17670	0.83	ukb-b-6811	1.48E-10	6.73E-05	76.61328084
rs1028455	ukb-b-17670	0.00700003	ukb-b-969	0.020576294	6.20E-05	33.22758573
rs10984444	ukb-b-17670	0.29	ukb-b-969	0.000226968	6.90E-05	37.08903594
rs11776021	ukb-b-17670	0.53	ukb-b-969	3.00E-05	7.50E-05	40.40967093
rs117799466	ukb-b-17670	0.77	ukb-b-969	8.89E-05	6.47E-05	32.42097276
rs12203592	ukb-b-17670	0.39	ukb-b-969	9.08E-05	6.97E-05	38.7392073
rs13251020	ukb-b-17670	0.88	ukb-b-969	0.000110584	5.61E-05	30.03521053
rs1368551	ukb-b-17670	0.29	ukb-b-969	5.40E-07	0.000116163	62.66337319
rs139577	ukb-b-17670	0.21	ukb-b-969	0.000325786	7.15E-05	37.75576804
rs1449390	ukb-b-17670	0.15	ukb-b-969	3.44E-05	9.28E-05	50.04946835
rs2356278	ukb-b-17670	0.25	ukb-b-969	0.001141177	5.80E-05	31.29722932
rs2413639	ukb-b-17670	0.85	ukb-b-969	5.76E-05	6.12E-05	32.85339032
rs251033	ukb-b-17670	0.69	ukb-b-969	3.78E-06	8.51E-05	45.68659656
rs2647259	ukb-b-17670	0.94	ukb-b-969	1.77E-05	6.69E-05	36.03117207
rs324300	ukb-b-17670	0.58	ukb-b-969	4.38E-06	8.76E-05	47.18087289
rs35660964	ukb-b-17670	0.83	ukb-b-969	7.34E-05	6.02E-05	32.20011662
rs3740422	ukb-b-17670	0.0759994	ukb-b-969	0.001667017	6.78E-05	36.32347847
rs4344697	ukb-b-17670	0.21	ukb-b-969	0.000174802	7.53E-05	40.57265761
rs61083878	ukb-b-17670	0.015	ukb-b-969	0.020652281	5.66E-05	30.28776978

rs62379243	ukb-b-17670	0.0680002	ukb-b-969	0.003311475	6.24E-05	33.57473303
rs627685	ukb-b-17670	0.1	ukb-b-969	0.000668392	7.36E-05	39.00547611
rs644799	ukb-b-17670	0.18	ukb-b-969	6.55E-06	0.000104298	56.2717856
rs7029718	ukb-b-17670	0.48	ukb-b-969	4.52E-05	7.40E-05	39.783403
rs7191618	ukb-b-17670	0.00980009	ukb-b-969	0.009785246	6.74E-05	36.29767531
rs7195043	ukb-b-17670	0.93	ukb-b-969	2.20E-05	6.79E-05	35.3141269
rs7560588	ukb-b-17670	0.19	ukb-b-969	0.00127382	6.22E-05	32.34840935
rs7578811	ukb-b-17670	0.83	ukb-b-969	0.000140695	5.52E-05	29.81444767
rs7587930	ukb-b-17670	0.57	ukb-b-969	1.47E-06	9.67E-05	51.72129048
rs7773004	ukb-b-17670	0.13	ukb-b-969	8.13E-05	8.77E-05	47.12603879
rs7852747	ukb-b-17670	0.2	ukb-b-969	0.000407425	6.89E-05	37.05719993
rs837065	ukb-b-17670	0.43	ukb-b-969	5.93E-05	7.39E-05	39.56900777
rs9319835	ukb-b-17670	0.13	ukb-b-969	0.00233728	5.98E-05	31.90499898
rs9427232	ukb-b-17670	0.12	ukb-b-969	0.003099505	5.70E-05	30.74498513
rs9508711	ukb-b-17670	0.2	ukb-b-969	0.00104176	6.14E-05	32.99984499
rs9852529	ukb-b-17670	0.52	ukb-b-969	1.24E-07	0.000115944	62.54943684

Supplementary Table 2: Summary of the study included in this two-sample mendelian randomization study.

id.exposure	id.outcome	method	b	se	pval	lo_ci	up_ci	or	or_lci95	or_uci95
ukb-b-151	ukb-b-17670	Inverse variance weighted	-0.002469209	0.001605097	0.123961851	-0.0056152	0.000676781	0.997533837	0.994400536	1.00067701
ukb-b-151	ukb-b-17670	MR Egger	-0.022205228	0.020789318	0.320916466	-0.06295229	0.018541835	0.978039494	0.938988271	1.018714802
ukb-b-151	ukb-b-17670	Weighted median	-0.002201755	0.00211864	0.29869755	-0.00635429	0.001950779	0.997800667	0.993665856	1.001952683
ukb-b-151	ukb-b-17670	Weighted mode	-0.002528871	0.003035017	0.428887785	-0.008477506	0.003419763	0.997474324	0.991558327	1.003425617
ukb-b-1553	ukb-b-17670	Inverse variance weighted	-0.004017909	0.049566458	0.935393402	-0.101168167	0.093132349	0.995990152	0.903781034	1.097606993
ukb-b-3793	ukb-b-17670	Inverse variance weighted	0.007314009	0.005787233	0.206295354	-0.004028967	0.018656985	1.007340822	0.995979138	1.018832114
ukb-b-3793	ukb-b-17670	MR Egger	0.116152588	0.086327514	0.310716329	-0.053049339	0.285354516	1.123167241	0.948333221	1.330233534
ukb-b-3793	ukb-b-17670	Weighted median	0.011170436	0.006669037	0.093940162	-0.001900877	0.024241749	1.011233058	0.998100928	1.024537969
ukb-b-3793	ukb-b-17670	Weighted mode	0.012829408	0.009454934	0.267883679	-0.005702263	0.031361079	1.012912058	0.994313964	1.031858019
ukb-b-4000	ukb-b-17670	Inverse variance weighted	0.010167902	0.00972635	0.295839289	-0.008895744	0.029231548	1.010219771	0.991143706	1.029662983
ukb-b-4000	ukb-b-17670	MR Egger	0.204480347	0.195509208	0.354650034	-0.178717702	0.587678395	1.226887343	0.836341964	1.799805125
ukb-b-4000	ukb-b-17670	Weighted	0.012121313	0.012058034	0.314777508	-0.011512433	0.035755059	1.012195074	0.988553581	1.036401958

		median								
ukb-b-4000	ukb-b-17670	Weighted mode	0.028813253	0.020674271	0.222190512	-0.011708318	0.069334823	1.02923237	0.988359958	1.071795011
ukb-b-4077	ukb-b-17670	Inverse variance weighted	0.002191635	0.014628566	0.880907561	-0.026480355	0.030863624	1.002194038	0.973867175	1.031344844
ukb-b-4077	ukb-b-17670	MR Egger	0.144928946	0.23595774	0.649345569	-0.317548225	0.607406116	1.155957432	0.727931576	1.83566372
ukb-b-4077	ukb-b-17670	Weighted median	-0.005862634	0.017796463	0.741833033	-0.040743701	0.029018434	0.994154518	0.960075164	1.029443571
ukb-b-4077	ukb-b-17670	Weighted mode	-0.008107543	0.021518002	0.742556842	-0.050282827	0.034067741	0.991925234	0.950960429	1.034654693
ukb-b-4171	ukb-b-17670	Inverse variance weighted	-0.007000383	0.007483344	0.349550286	-0.021667737	0.00766697	0.993024062	0.978565322	1.007696437
ukb-b-4171	ukb-b-17670	MR Egger	-0.046363271	0.060497192	0.461163769	-0.164937769	0.072211226	0.954695086	0.847946471	1.074882363
ukb-b-4171	ukb-b-17670	Weighted median	-0.001194059	0.009478218	0.899748322	-0.019771366	0.017383248	0.998806653	0.980422805	1.017535216
ukb-b-4171	ukb-b-17670	Weighted mode	0.001634988	0.016302949	0.921920268	-0.030318792	0.033588769	1.001636326	0.970136213	1.034159241
ukb-b-4522	ukb-b-17670	Inverse variance weighted	0.001881072	0.001506736	0.211869406	-0.001072131	0.004834274	1.001882842	0.998928444	1.004845978
ukb-b-4522	ukb-b-17670	MR Egger	0.006875286	0.011207086	0.541735462	-0.015090602	0.028841174	1.006898975	0.985022691	1.029261108
ukb-b-4522	ukb-b-17670	Weighted median	0.001852833	0.002085057	0.374204756	-0.002233878	0.005939545	1.001854551	0.997768615	1.005957219
ukb-b-4522	ukb-b-17670	Weighted	0.005439376	0.005539665	0.329791685	-0.005418367	0.016297119	1.005454196	0.994596286	1.016430641

		mode								
ukb-b-4667	ukb-b-17670	Inverse variance weighted	0.006456664	0.006939117	0.352126033	-0.007144006	0.020057334	1.006477553	0.992881452	1.020259834
ukb-b-4667	ukb-b-17670	MR Egger	-0.072641507	0.080887459	0.38434026	-0.231180926	0.085897912	0.929934145	0.793595871	1.089695078
ukb-b-4667	ukb-b-17670	Weighted median	0.009323071	0.009587113	0.330822454	-0.009467671	0.028113813	1.009366666	0.990577006	1.028512736
ukb-b-4667	ukb-b-17670	Weighted mode	0.015216008	0.018402733	0.421289829	-0.020853348	0.051285364	1.015332361	0.97936258	1.052623231
ukb-b-4710	ukb-b-17670	Inverse variance weighted	-0.002006323	0.001446232	0.165357768	-0.004840938	0.000828293	0.997995689	0.995170761	1.000828636
ukb-b-4710	ukb-b-17670	MR Egger	0.011308652	0.020968299	0.601465803	-0.029789215	0.052406519	1.011372836	0.97065011	1.053804047
ukb-b-4710	ukb-b-17670	Weighted median	-0.001507823	0.001708962	0.377612795	-0.00485739	0.001841743	0.998493313	0.995154389	1.00184344
ukb-b-4710	ukb-b-17670	Weighted mode	0.000944265	0.003340656	0.782686536	-0.00560342	0.00749195	1.000944711	0.99441225	1.007520085
ukb-b-4886	ukb-b-17670	Inverse variance weighted	0.000274111	0.001451929	0.850256792	-0.002571669	0.003119891	1.000274148	0.997431635	1.003124763
ukb-b-4886	ukb-b-17670	MR Egger	0.021446932	0.016370686	0.209876925	-0.010639613	0.053533477	1.02167857	0.989416787	1.054992309
ukb-b-4886	ukb-b-17670	Weighted median	-0.00127033	0.001786431	0.477022749	-0.004771736	0.002231076	0.998730477	0.995239631	1.002233566
ukb-b-4886	ukb-b-17670	Weighted mode	-0.003291128	0.004456518	0.470906923	-0.012025903	0.005443648	0.996714282	0.988046119	1.005458491
ukb-b-5076	ukb-b-17670	Inverse	0.002043125	0.009271799	0.825591543	-0.016129601	0.020215851	1.002045213	0.983999784	1.020421575

		variance weighted								
ukb-b-5076	ukb-b-17670	MR Egger	0.07268867	0.070537009	0.342511219	-0.065563868	0.210941209	1.075395682	0.93653923	1.234839755
ukb-b-5076	ukb-b-17670	Weighted median	-0.006413956	0.010613305	0.545623178	-0.027216034	0.014388122	0.993606569	0.973150985	1.014492129
ukb-b-5076	ukb-b-17670	Weighted mode	-0.012104646	0.017357467	0.508061641	-0.046125282	0.02191599	0.987968321	0.95492232	1.02215791
ukb-b-5192	ukb-b-17670	Inverse variance weighted	0.00024607	0.00151049	0.870591195	-0.002714491	0.003206632	1.000246101	0.99728919	1.003211778
ukb-b-5192	ukb-b-17670	MR Egger	-0.005175124	0.008668194	0.55226552	-0.022164784	0.011814536	0.994838244	0.97807905	1.011884603
ukb-b-5192	ukb-b-17670	Weighted median	-0.000360519	0.002071576	0.861840902	-0.004420808	0.00369977	0.999639546	0.995588949	1.003706623
ukb-b-5192	ukb-b-17670	Weighted mode	8.77E-05	0.00434961	0.983973581	-0.008437579	0.008612893	1.000087661	0.991597917	1.008650091
ukb-b-6811	ukb-b-17670	Inverse variance weighted	-0.007757324	0.007413048	0.295357042	-0.022286898	0.00677225	0.992272686	0.97795962	1.006795233
ukb-b-6811	ukb-b-17670	MR Egger	0.021852611	0.05357285	0.753435862	-0.083150175	0.126855397	1.022093128	0.920212944	1.135252845
ukb-b-6811	ukb-b-17670	Weighted median	-0.005462493	0.008213	0.505984453	-0.021559973	0.010634987	0.994552399	0.978670782	1.010691739
ukb-b-6811	ukb-b-17670	Weighted mode	-0.003646192	0.010247919	0.756015776	-0.023732113	0.016439728	0.996360447	0.976547279	1.016575604
ukb-b-969	ukb-b-17670	Inverse variance weighted	-0.00536955	0.002096257	0.010422294	-0.009478213	-0.001260887	0.994644841	0.990566564	0.998739908

ukb-b-969	ukb-b-17670	MR Egger	-0.018032552	0.020159903	0.377745931	-0.057545961	0.021480857	0.982129062	0.944078498	1.021713232
ukb-b-969	ukb-b-17670	Weighted median	-0.005241269	0.002624845	0.045847804	-0.010385964	-9.66E-05	0.994772443	0.989667783	0.999903432
ukb-b-969	ukb-b-17670	Weighted mode	-0.007050224	0.005117536	0.177580252	-0.017080594	0.002980147	0.992974571	0.983064452	1.002984592

Supplementary Table 3: Summary of the leave–one–out sensitivity included in this two-sample mendelian randomization study.

id.exposure	id.outcome	sample size	SNP	b	se	p
ukb-b-151	ukb-b-17670	462933	rs1491872	-0.002550559	0.00168740848623564	0.130654889533478
ukb-b-151	ukb-b-17670	462933	rs2005617	-0.002789408	0.00168914822375817	0.0986631324448669
ukb-b-151	ukb-b-17670	462933	rs2189464	-0.002516282	0.00168037417494802	0.134275259937632
ukb-b-151	ukb-b-17670	462933	rs2764261	-0.002283582	0.00171122408092193	0.182048962677925
ukb-b-151	ukb-b-17670	462933	rs328900	-0.001569607	0.00171156100354537	0.359110258968359
ukb-b-151	ukb-b-17670	462933	rs382210	-0.002756105	0.00170945939894743	0.106903818465576
ukb-b-151	ukb-b-17670	462933	rs6533635	-0.002465834	0.00169307399891077	0.145275379971855
ukb-b-151	ukb-b-17670	462933	rs6955240	-0.00215447	0.00175280881507595	0.219014438606652
ukb-b-151	ukb-b-17670	462933	rs7072776	-0.003093479	0.00169044108537171	0.0672524553131139
ukb-b-151	ukb-b-17670	462933	All	-0.002469209	0.00160509722507043	0.123961850764123
ukb-b-1553	ukb-b-17670	462933	All	-0.004017909	0.0495664581865704	0.93539340183772
ukb-b-3793	ukb-b-17670	462933	rs11057408	0.00537833395323825	0.00665871053337231	0.419255222157454
ukb-b-3793	ukb-b-17670	462933	rs12069474	0.00541395985232564	0.00658487669103214	0.410973946899612
ukb-b-3793	ukb-b-17670	462933	rs12921753	0.00635863704482924	0.00726956084579284	0.381740740818209
ukb-b-3793	ukb-b-17670	462933	rs2588917	0.0119836311895809	0.00659132896003276	0.0690503336338443
ukb-b-3793	ukb-b-17670	462933	All	0.00731400909146736	0.00578723281624392	0.206295353511517
ukb-b-4000	ukb-b-17670	462933	rs11712056	0.0130844380990384	0.0116651545186953	0.262003351599837
ukb-b-4000	ukb-b-17670	462933	rs13002862	0.00548279066759896	0.0101966832639381	0.590781871683471
ukb-b-4000	ukb-b-17670	462933	rs2499760	0.0146523833937098	0.0103980991257061	0.158793604773209
ukb-b-4	ukb-b-1	462933	rs62263	0.005193411890	0.01067966752	0.62676180940

000	7670		912	59317	93585	1516
ukb-b-4000	ukb-b-17670	462933	rs6478444	0.00722299575269289	0.0111853994169967	0.518439879399876
ukb-b-4000	ukb-b-17670	462933	rs7228990	0.0152975251495624	0.0101031443389423	0.129991585835088
ukb-b-4000	ukb-b-17670	462933	All	0.0101679019765383	0.00972634994999496	0.295839289082347
ukb-b-4077	ukb-b-17670	462933	rs11917431	-0.008129959	0.0182033577009174	0.655150425795477
ukb-b-4077	ukb-b-17670	462933	rs17824247	0.00698625698028426	0.0175578990888065	0.690705204377269
ukb-b-4077	ukb-b-17670	462933	rs2773485	0.00724815556284537	0.0180059995358196	0.687285818427732
ukb-b-4077	ukb-b-17670	462933	All	0.00219163452397285	0.0146285661920065	0.880907560796502
ukb-b-4171	ukb-b-17670	462933	rs10978543	-0.00866145	0.0079346519721573	0.275009865673735
ukb-b-4171	ukb-b-17670	462933	rs12103006	-0.007223951	0.00811411080316894	0.373307556991117
ukb-b-4171	ukb-b-17670	462933	rs12472555	-0.007748963	0.00813441453288879	0.340785346364712
ukb-b-4171	ukb-b-17670	462933	rs12759477	-0.008032346	0.00830925534497853	0.333706714238135
ukb-b-4171	ukb-b-17670	462933	rs139920	-0.008756089	0.00808441077185181	0.278771511249575
ukb-b-4171	ukb-b-17670	462933	rs2661863	-0.006171179	0.00807070423107072	0.444486268564637
ukb-b-4171	ukb-b-17670	462933	rs36030660	-0.00598222	0.00806491988907653	0.458233914053801
ukb-b-4171	ukb-b-17670	462933	rs61873510	-0.004201267	0.00751185155347069	0.575967156403213
ukb-b-4171	ukb-b-17670	462933	rs6969458	-0.003225514	0.00778610516543035	0.678679722236016
ukb-b-4171	ukb-b-17670	462933	rs754204	-0.009150961	0.0076876463456411	0.233910307836311
ukb-b-4171	ukb-b-17670	462933	rs780094	-0.003865249	0.00716983500790613	0.589818723752058
ukb-b-4171	ukb-b-17670	462933	rs9401593	-0.010847645	0.00682553416310292	0.111998522462303
ukb-b-4171	ukb-b-17670	462933	All	-0.007000383	0.00748334362247553	0.349550285880847
ukb-b-4522	ukb-b-17670	462933	rs10208088	0.00200491948193433	0.00152080969332681	0.187395306098863

ukb-b-4522	ukb-b-17670	462933	rs1037091	0.00188673621617157	0.00153654100899772	0.219480230861333
ukb-b-4522	ukb-b-17670	462933	rs10518019	0.00152113358584265	0.00146816331150715	0.300165158048989
ukb-b-4522	ukb-b-17670	462933	rs10828248	0.00193358753222302	0.00152636204967705	0.205228666128335
ukb-b-4522	ukb-b-17670	462933	rs11634155	0.00212388954848777	0.00150683286975331	0.158685683391584
ukb-b-4522	ukb-b-17670	462933	rs11652437	0.00203778769808386	0.00152305094510708	0.180908094645807
ukb-b-4522	ukb-b-17670	462933	rs117405403	0.00211853850388451	0.00151106510167649	0.160910260531536
ukb-b-4522	ukb-b-17670	462933	rs11749912	0.0016088817690612	0.00151233309676589	0.287400789756521
ukb-b-4522	ukb-b-17670	462933	rs11766392	0.00166944435665643	0.00151510904953075	0.270520732935205
ukb-b-4522	ukb-b-17670	462933	rs11942953	0.00201573349061724	0.00152053853023687	0.18494877204939
ukb-b-4522	ukb-b-17670	462933	rs12128707	0.0017096999963592	0.00151670390896435	0.259638045761931
ukb-b-4522	ukb-b-17670	462933	rs12145677	0.00211104638280123	0.00152500270794316	0.166269597669183
ukb-b-4522	ukb-b-17670	462933	rs12521638	0.00198120410073343	0.00152289852474081	0.19327798287941
ukb-b-4522	ukb-b-17670	462933	rs12553324	0.0017849699643295	0.0015437845842921	0.247587114559789
ukb-b-4522	ukb-b-17670	462933	rs12706626	0.00188387217358586	0.00152751235319136	0.217466009317324
ukb-b-4522	ukb-b-17670	462933	rs12820967	0.00184467106728519	0.00152878101909474	0.227575165413218
ukb-b-4522	ukb-b-17670	462933	rs12946454	0.00172519688886042	0.00151965867086971	0.256269377235995
ukb-b-4522	ukb-b-17670	462933	rs13262595	0.00143406616002445	0.00150322372681774	0.340086765720773
ukb-b-4522	ukb-b-17670	462933	rs136553	0.00205892564519125	0.0015173784924541	0.174814082440629
ukb-b-4522	ukb-b-17670	462933	rs1395020	0.0019736437781498	0.00152369663755113	0.195216867040497
ukb-b-4522	ukb-b-17670	462933	rs1448355	0.00202884999793675	0.00152410594364182	0.183131809149833
ukb-b-4522	ukb-b-17670	462933	rs1469249	0.00188616974156467	0.00152827092732236	0.217133808064407
ukb-b-4522	ukb-b-17670	462933	rs1648906	0.00167113382830193	0.00150906374112953	0.268121987762121

ukb-b-4522	ukb-b-17670	462933	rs166835	0.00189573340442054	0.00152857222482971	0.214901880501623
ukb-b-4522	ukb-b-17670	462933	rs17167210	0.00176826791177435	0.00152538013813815	0.246362046873159
ukb-b-4522	ukb-b-17670	462933	rs17789218	0.00199771386780707	0.00152400590129371	0.189914935801854
ukb-b-4522	ukb-b-17670	462933	rs17862355	0.00170298417856742	0.00151812920359078	0.261962379010583
ukb-b-4522	ukb-b-17670	462933	rs1987942	0.00158410912786142	0.00149375910689458	0.2889240100782
ukb-b-4522	ukb-b-17670	462933	rs1999244	0.00186339371123642	0.00152868826325957	0.222863380883351
ukb-b-4522	ukb-b-17670	462933	rs2032780	0.00175649860366909	0.00152651193608857	0.249871497079854
ukb-b-4522	ukb-b-17670	462933	rs2041687	0.00191901391323921	0.00152956066768955	0.20961756865158
ukb-b-4522	ukb-b-17670	462933	rs2068625	0.0021051423007445	0.00152035055062209	0.166161746125196
ukb-b-4522	ukb-b-17670	462933	rs206965	0.00168746184119903	0.00151326197915983	0.264800943558625
ukb-b-4522	ukb-b-17670	462933	rs2120461	0.00178110521673305	0.00152698448489401	0.243444683090029
ukb-b-4522	ukb-b-17670	462933	rs2220599	0.00223276331699532	0.00149145488172427	0.134383600268511
ukb-b-4522	ukb-b-17670	462933	rs246723	0.0021787697124208	0.00149140988456551	0.14404858959382
ukb-b-4522	ukb-b-17670	462933	rs2588543	0.00183871091317383	0.00152693685363706	0.228518978647045
ukb-b-4522	ukb-b-17670	462933	rs2734833	0.00223229853876798	0.00149361607979238	0.135029366099318
ukb-b-4522	ukb-b-17670	462933	rs2748985	0.00172198370088307	0.00152495040400592	0.258810789749058
ukb-b-4522	ukb-b-17670	462933	rs2756121	0.0014887163431246	0.00147326321561101	0.312261044286018
ukb-b-4522	ukb-b-17670	462933	rs2761438	0.00201560225066548	0.00152252707178823	0.185551822305845
ukb-b-4522	ukb-b-17670	462933	rs28710456	0.00202988908842846	0.00151894727764075	0.181425460157103
ukb-b-4522	ukb-b-17670	462933	rs306755	0.00213592779784885	0.001500928994251	0.154715714527652
ukb-b-4522	ukb-b-17670	462933	rs422115	0.00179542582898637	0.00152550066201651	0.239218649322524
ukb-b-4522	ukb-b-17670	462933	rs4704043	0.00193591764111266	0.0015270217312493	0.204878858467718

ukb-b-4522	ukb-b-17670	462933	rs4852252	0.00174257676713374	0.00151917317051528	0.251358459009297
ukb-b-4522	ukb-b-17670	462933	rs56229818	0.00185576331279795	0.00152726419450644	0.224331801449967
ukb-b-4522	ukb-b-17670	462933	rs58638214	0.00190631215762867	0.00153224102740907	0.213450489809647
ukb-b-4522	ukb-b-17670	462933	rs6028090	0.0020244053600713	0.00152614469617561	0.184679683269776
ukb-b-4522	ukb-b-17670	462933	rs6449708	0.00187968854599525	0.00152827711919978	0.218719724387407
ukb-b-4522	ukb-b-17670	462933	rs6780848	0.0017804982176207	0.00152304986856227	0.24238963219727
ukb-b-4522	ukb-b-17670	462933	rs6935828	0.00205635816779413	0.00151414906816694	0.174433564814932
ukb-b-4522	ukb-b-17670	462933	rs7020477	0.00187004895525058	0.00152831567045674	0.221102690189481
ukb-b-4522	ukb-b-17670	462933	rs7209653	0.00149156471164963	0.00148627078450891	0.315589833461214
ukb-b-4522	ukb-b-17670	462933	rs7281293	0.00204298854025679	0.00151898066443383	0.178633835112383
ukb-b-4522	ukb-b-17670	462933	rs7288455	0.00172356216479237	0.00151801324847198	0.256205022205529
ukb-b-4522	ukb-b-17670	462933	rs73578186	0.00172986608423311	0.00152233779547931	0.255821783873145
ukb-b-4522	ukb-b-17670	462933	rs7526112	0.00215260632442129	0.00149780202313385	0.150667762259242
ukb-b-4522	ukb-b-17670	462933	rs7564844	0.00197082481333679	0.00152702277272623	0.196831232937001
ukb-b-4522	ukb-b-17670	462933	rs7630869	0.00174122123125272	0.00153428464650333	0.256427711625591
ukb-b-4522	ukb-b-17670	462933	rs7904398	0.00182409876740093	0.00152626945411064	0.232034104126792
ukb-b-4522	ukb-b-17670	462933	rs7968738	0.00181808827142423	0.00152749578856112	0.233951642477362
ukb-b-4522	ukb-b-17670	462933	rs79720045	0.00176422750135869	0.00152782416377701	0.248200177889852
ukb-b-4522	ukb-b-17670	462933	rs806795	0.00193636772681447	0.00152669783054369	0.204677540265542
ukb-b-4522	ukb-b-17670	462933	rs8102851	0.00180767141536684	0.00152620550448625	0.236246045556578
ukb-b-4522	ukb-b-17670	462933	rs9375188	0.00234990198703199	0.00150171500424655	0.117626946005185
ukb-b-4522	ukb-b-17670	462933	All	0.00188107162576018	0.00150673585562253	0.211869406080118

ukb-b-4667	ukb-b-17670	462933	rs12156017	0.0062826548684474	0.00716958602922245	0.380871010708047
ukb-b-4667	ukb-b-17670	462933	rs12992090	0.00941736148516976	0.00720962182345227	0.191477316351565
ukb-b-4667	ukb-b-17670	462933	rs1471093	0.00912933293825594	0.007257223590528	0.208404431075652
ukb-b-4667	ukb-b-17670	462933	rs17527878	0.00557349091133802	0.0071505891336414	0.435717627416699
ukb-b-4667	ukb-b-17670	462933	rs1991083	0.00915567380395011	0.00714576842372482	0.20009810463911
ukb-b-4667	ukb-b-17670	462933	rs3197999	0.00630700979271479	0.00714173772210792	0.3771715621593
ukb-b-4667	ukb-b-17670	462933	rs332828	0.00614614486208062	0.00724158684878012	0.396032097987188
ukb-b-4667	ukb-b-17670	462933	rs36104984	0.00598318404492224	0.00713123019175029	0.401462863795662
ukb-b-4667	ukb-b-17670	462933	rs410671	0.00417828228039176	0.00722966691020239	0.563307562077562
ukb-b-4667	ukb-b-17670	462933	rs6545977	0.00481951231624825	0.00712522305093947	0.498785697744114
ukb-b-4667	ukb-b-17670	462933	rs6722794	0.00701353640281953	0.00713061208711421	0.325321444282294
ukb-b-4667	ukb-b-17670	462933	rs6862251	0.00857379653694977	0.00718618311596083	0.232832311597603
ukb-b-4667	ukb-b-17670	462933	rs699534	0.00448776576887788	0.00717869870501496	0.531872406589304
ukb-b-4667	ukb-b-17670	462933	rs7388625	0.00555347821386074	0.00712010717935013	0.435407854360363
ukb-b-4667	ukb-b-17670	462933	rs8020432	0.00686530659501861	0.00711606893226874	0.334664476658543
ukb-b-4667	ukb-b-17670	462933	rs990702	0.00391308369927219	0.0071396208781256	0.583636945006435
ukb-b-4667	ukb-b-17670	462933	All	0.00645666402933374	0.00693911729405777	0.352126032752534
ukb-b-4710	ukb-b-17670	462933	rs10098073	-0.001357566	0.00143214085474375	0.343166105081789
ukb-b-4710	ukb-b-17670	462933	rs1036800	-0.002630981	0.00139139773631638	0.0586389650808008
ukb-b-4710	ukb-b-17670	462933	rs11749912	-0.001564249	0.0014986138266488	0.296579227542739
ukb-b-4710	ukb-b-17670	462933	rs2246122	-0.002375536	0.00152061828177557	0.11823685321683
ukb-b-4710	ukb-b-17670	462933	rs404907	-0.002282226	0.001565556525462	0.144903182383227

ukb-b-4710	ukb-b-17670	462933	rs4129572	-0.001699076	0.00158771430738169	0.284556536164594
ukb-b-4710	ukb-b-17670	462933	rs4540651	-0.00203936	0.00157741527133887	0.196063300475847
ukb-b-4710	ukb-b-17670	462933	rs4886868	-0.002533703	0.00147695497787789	0.0862552135981427
ukb-b-4710	ukb-b-17670	462933	rs7229874	-0.002414813	0.00150371256019758	0.108295685586222
ukb-b-4710	ukb-b-17670	462933	rs7565480	-0.001464381	0.00144796841692745	0.311856167206942
ukb-b-4710	ukb-b-17670	462933	rs9533455	-0.00206412	0.00157898585779022	0.191129795707725
ukb-b-4710	ukb-b-17670	462933	rs997467	-0.001628045	0.00153740704127807	0.289620113651006
ukb-b-4710	ukb-b-17670	462933	All	-0.002006323	0.00144623222515758	0.165357767801028
ukb-b-4886	ukb-b-17670	462933	rs11183184	6.51031340237152e-05	0.0015207259769949	0.965852544293767
ukb-b-4886	ukb-b-17670	462933	rs12568280	0.000614836413286132	0.0014993736504344	0.68176034222581
ukb-b-4886	ukb-b-17670	462933	rs12979056	0.000888669102045217	0.00138890528407991	0.522280395409322
ukb-b-4886	ukb-b-17670	462933	rs1455343	0.00047410855973811	0.00154372329842869	0.758751906730647
ukb-b-4886	ukb-b-17670	462933	rs2011071	-4.60E-05	0.00149469537302257	0.975423762412964
ukb-b-4886	ukb-b-17670	462933	rs2220599	-0.000378399	0.00143565290283628	0.792108945108211
ukb-b-4886	ukb-b-17670	462933	rs34654885	6.85845078084413e-05	0.00153466753423741	0.964354292546042
ukb-b-4886	ukb-b-17670	462933	rs4588066	0.000618083642394178	0.00148538115421276	0.677328680132372
ukb-b-4886	ukb-b-17670	462933	rs4641022	0.000396814341308136	0.00153150757767106	0.795557718078263
ukb-b-4886	ukb-b-17670	462933	rs62172117	0.000311575477256803	0.00155251513227632	0.840940367322628
ukb-b-4886	ukb-b-17670	462933	rs62422661	-0.000256711	0.00147513260875696	0.861845515463973
ukb-b-4886	ukb-b-17670	462933	rs62482241	-0.000168639	0.00146694125032932	0.908477111917995
ukb-b-4886	ukb-b-17670	462933	rs627685	0.000870865386466379	0.00144624928436528	0.547071358483713
ukb-b-4886	ukb-b-17670	462933	rs72931435	0.000464153890087053	0.00152023614567586	0.7601247542132

ukb-b-4886	ukb-b-17670	462933	rs747344	0.000449299766229963	0.00152247951572689	0.76790943122348
ukb-b-4886	ukb-b-17670	462933	rs7576657	-0.000161822	0.00148355608159027	0.913141205074581
ukb-b-4886	ukb-b-17670	462933	rs9400239	0.000428845556720395	0.0015255846317744	0.778631832612415
ukb-b-4886	ukb-b-17670	462933	All	0.000274110726783173	0.00145192850705391	0.850256792221014
ukb-b-5076	ukb-b-17670	462933	rs13011181	-0.00263893	0.0100197690145083	0.792263609279991
ukb-b-5076	ukb-b-17670	462933	rs2043145	0.00334008776663456	0.0105830716568904	0.75230109137131
ukb-b-5076	ukb-b-17670	462933	rs2675638	-5.60E-05	0.0103147831283358	0.995672031520903
ukb-b-5076	ukb-b-17670	462933	rs3197999	0.00349847014634635	0.0107593447573329	0.745062681155478
ukb-b-5076	ukb-b-17670	462933	rs35291206	0.00480123579984466	0.0100504935543825	0.632855597163681
ukb-b-5076	ukb-b-17670	462933	rs410671	0.00620823209127624	0.00931043181247736	0.504897411702826
ukb-b-5076	ukb-b-17670	462933	rs4247450	-0.003718543	0.0082772899602494	0.653253882306514
ukb-b-5076	ukb-b-17670	462933	rs847687	0.00464684272905472	0.010114795363514	0.645939442603659
ukb-b-5076	ukb-b-17670	462933	All	0.00204312473960548	0.00927179908510673	0.825591543194002
ukb-b-5192	ukb-b-17670	462933	rs10109061	0.00022894360895045	0.00152727288446221	0.88084072030008
ukb-b-5192	ukb-b-17670	462933	rs10189857	0.000551253461571004	0.0015236790736259	0.71750817607203
ukb-b-5192	ukb-b-17670	462933	rs10269099	0.000164654321639372	0.00152453558574378	0.913993362025204
ukb-b-5192	ukb-b-17670	462933	rs10739499	0.000121024911814626	0.0015197462712887	0.93652760080504
ukb-b-5192	ukb-b-17670	462933	rs10765777	0.000508823822755227	0.00151045205874565	0.736215980578997
ukb-b-5192	ukb-b-17670	462933	rs11191129	0.000376461096212987	0.00152242591432453	0.804693764295333
ukb-b-5192	ukb-b-17670	462933	rs11245482	0.000143469907080084	0.00152536033427028	0.925064351762721
ukb-b-5192	ukb-b-17670	462933	rs11662211	0.000572957512285363	0.00148420478227781	0.699469490825347
ukb-b-5192	ukb-b-17670	462933	rs11680095	0.000391681987973663	0.00151788952676432	0.796373100738322

ukb-b-5192	ukb-b-17670	462933	rs11700249	0.00039175074302126	0.00151910682591197	0.796497755259934
ukb-b-5192	ukb-b-17670	462933	rs11714337	0.000174740748729573	0.00152712270028517	0.908901035213785
ukb-b-5192	ukb-b-17670	462933	rs11877758	0.000326377976406144	0.00152761608265064	0.830818575524274
ukb-b-5192	ukb-b-17670	462933	rs11911112	0.000400894786791372	0.00152193809242426	0.792234040023273
ukb-b-5192	ukb-b-17670	462933	rs12214364	0.00036340180826083	0.00152072746106327	0.811132125659519
ukb-b-5192	ukb-b-17670	462933	rs12553324	0.000378739764186343	0.00153309972480112	0.804875850149532
ukb-b-5192	ukb-b-17670	462933	rs1291871	0.00028232477124325	0.00152734091473189	0.853348844541533
ukb-b-5192	ukb-b-17670	462933	rs13014947	0.000150675693392729	0.00152708014013575	0.921400967167691
ukb-b-5192	ukb-b-17670	462933	rs1727332	0.00085644813219857	0.00142234103390095	0.547081089946289
ukb-b-5192	ukb-b-17670	462933	rs17789218	0.000169593635972137	0.00152596740427214	0.911506567260862
ukb-b-5192	ukb-b-17670	462933	rs178203	0.000254141427658236	0.00153021970357361	0.86809268735819
ukb-b-5192	ukb-b-17670	462933	rs180396	0.000430269106621032	0.00151090650592301	0.775816168411517
ukb-b-5192	ukb-b-17670	462933	rs1993092	0.000232769634229486	0.00152812040797942	0.878931282362086
ukb-b-5192	ukb-b-17670	462933	rs2106164	0.000393211359356962	0.00151923340970691	0.795772415075295
ukb-b-5192	ukb-b-17670	462933	rs2185490	0.000179213009351115	0.00152584942404349	0.906502428165016
ukb-b-5192	ukb-b-17670	462933	rs2283	0.000430967990504498	0.00151117855499905	0.775501126917772
ukb-b-5192	ukb-b-17670	462933	rs2332818	0.000286024414432547	0.00152654766084002	0.851373018409143
ukb-b-5192	ukb-b-17670	462933	rs2352984	0.000283823002367636	0.00155590198090033	0.855255470065903
ukb-b-5192	ukb-b-17670	462933	rs262890	0.000227645084524945	0.0015336517352472	0.882000771932239
ukb-b-5192	ukb-b-17670	462933	rs263771	0.000292141407393965	0.00152890985077051	0.848464293450545
ukb-b-5192	ukb-b-17670	462933	rs2646351	0.000415463879217395	0.00151412531588532	0.783783387440248
ukb-b-5192	ukb-b-17670	462933	rs2678662	0.000325620823831836	0.00152910788205903	0.831367339416341

ukb-b-5192	ukb-b-17670	462933	rs2725371	-9.12E-05	0.00150563200541015	0.951714981336283
ukb-b-5192	ukb-b-17670	462933	rs2906604	0.000159203172626202	0.00152769584273689	0.917001665216881
ukb-b-5192	ukb-b-17670	462933	rs3138499	0.000220970383686915	0.00153090928920622	0.885232541908833
ukb-b-5192	ukb-b-17670	462933	rs34094119	0.000262908399239325	0.00152767267796767	0.863361006525853
ukb-b-5192	ukb-b-17670	462933	rs34811474	0.000170018101422687	0.00152983402477483	0.911509296168272
ukb-b-5192	ukb-b-17670	462933	rs35797019	0.000295949152281789	0.00152659631327004	0.84628386809833
ukb-b-5192	ukb-b-17670	462933	rs362312	0.000147663998848745	0.00152436868906574	0.922830464765117
ukb-b-5192	ukb-b-17670	462933	rs3754970	0.00025041841808299	0.00152880827394574	0.869888785910869
ukb-b-5192	ukb-b-17670	462933	rs3810496	2.45408604794718e-05	0.00150592014906189	0.986998044311265
ukb-b-5192	ukb-b-17670	462933	rs4076457	4.98760116496345e-06	0.00149949907690296	0.997346098648289
ukb-b-5192	ukb-b-17670	462933	rs4110177	9.87079780254044e-05	0.00151808969081363	0.948157139366092
ukb-b-5192	ukb-b-17670	462933	rs4303732	6.37496959925484e-05	0.00151943695544419	0.966533669667048
ukb-b-5192	ukb-b-17670	462933	rs4339469	1.35894055139994e-05	0.00152025994425742	0.992867908875042
ukb-b-5192	ukb-b-17670	462933	rs4469687	-0.000122976	0.00146172654048267	0.932952311305855
ukb-b-5192	ukb-b-17670	462933	rs4747438	0.000286833343575189	0.00153095302197165	0.851381462673909
ukb-b-5192	ukb-b-17670	462933	rs4788616	0.000386833621779764	0.00152199251873767	0.799369919541868
ukb-b-5192	ukb-b-17670	462933	rs4847408	-7.18E-06	0.0015054308121446	0.996191957936204
ukb-b-5192	ukb-b-17670	462933	rs494566	0.000179665612087951	0.0015268510441935	0.906328593367992
ukb-b-5192	ukb-b-17670	462933	rs57555420	0.000510074653201491	0.00149704611815988	0.733313827704227
ukb-b-5192	ukb-b-17670	462933	rs6102912	0.000272234537106279	0.0015306634468207	0.858837678287418
ukb-b-5192	ukb-b-17670	462933	rs61864793	0.000223608557599159	0.00152865782442012	0.883702165719731
ukb-b-5192	ukb-b-17670	462933	rs62145951	0.000389247267882053	0.00152389172569117	0.798391202915893

ukb-b-5192	ukb-b-17670	462933	rs62199883	0.000220534107375972	0.00153774007939275	0.885962892214057
ukb-b-5192	ukb-b-17670	462933	rs6511708	0.000405814549013073	0.00152508044632638	0.790166920450069
ukb-b-5192	ukb-b-17670	462933	rs6814554	0.000305986145907477	0.00153624536632321	0.842123576144498
ukb-b-5192	ukb-b-17670	462933	rs6850494	9.25583301029939e-05	0.00151862731184054	0.951400080426859
ukb-b-5192	ukb-b-17670	462933	rs6994132	0.000156086060592584	0.00152611040427035	0.918536776170721
ukb-b-5192	ukb-b-17670	462933	rs7089973	0.000249232403302887	0.00152768235062993	0.870404956931454
ukb-b-5192	ukb-b-17670	462933	rs7184800	0.000149333773469556	0.00153213443387077	0.922354903863161
ukb-b-5192	ukb-b-17670	462933	rs749056	0.000104888808421635	0.00151944189432157	0.944964831277011
ukb-b-5192	ukb-b-17670	462933	rs749671	0.000185226922518654	0.00152965672451023	0.90361934080417
ukb-b-5192	ukb-b-17670	462933	rs7539775	0.000281464485354682	0.00152664561126715	0.853724829686337
ukb-b-5192	ukb-b-17670	462933	rs75499503	4.66245373960729e-05	0.00153480891598298	0.975765531940485
ukb-b-5192	ukb-b-17670	462933	rs7708324	0.000251104446387328	0.00152871720856383	0.869527829917285
ukb-b-5192	ukb-b-17670	462933	rs7798292	-0.000155664	0.00146421629015024	0.915334948285961
ukb-b-5192	ukb-b-17670	462933	rs7899206	3.29556071771299e-05	0.00151098847672276	0.982599016498123
ukb-b-5192	ukb-b-17670	462933	rs7921305	0.000460463774973553	0.00151244607161747	0.760785297320064
ukb-b-5192	ukb-b-17670	462933	rs801733	0.000291552195192559	0.0015329191959933	0.849157029995865
ukb-b-5192	ukb-b-17670	462933	rs814197	0.000232131702186696	0.00153063310942484	0.879457228149641
ukb-b-5192	ukb-b-17670	462933	rs883027	-9.53E-06	0.00150222426819461	0.994938377966186
ukb-b-5192	ukb-b-17670	462933	rs898751	0.000335625801349585	0.00152657792053263	0.825984077150396
ukb-b-5192	ukb-b-17670	462933	rs9300594	5.04760590906362e-06	0.00150653694215198	0.997326717204107
ukb-b-5192	ukb-b-17670	462933	rs9471333	0.00025929654197918	0.00153010555425686	0.86543233720126
ukb-b-5192	ukb-b-17670	462933	rs9834970	0.000254536588057412	0.00152755897989118	0.867661478035308

ukb-b-5192	ukb-b-17670	462933	rs9867437	0.000237034962141954	0.00152983680161508	0.876867563525167
ukb-b-5192	ukb-b-17670	462933	rs9880023	0.000366137045505375	0.00152284112549481	0.809996743485031
ukb-b-5192	ukb-b-17670	462933	rs996234	0.000393919891977285	0.00152204889992287	0.795782644732089
ukb-b-5192	ukb-b-17670	462933	All	0.000246070351557941	0.00151049040831531	0.870591195010634
ukb-b-6811	ukb-b-17670	462933	rs12956276	-0.00809868	0.00846351139885208	0.338620980047364
ukb-b-6811	ukb-b-17670	462933	rs1368549	-0.003703443	0.00882265334838511	0.674656944785393
ukb-b-6811	ukb-b-17670	462933	rs4580876	-0.012692558	0.0102130570623191	0.213949819430508
ukb-b-6811	ukb-b-17670	462933	All	-0.007757324	0.00741304777347018	0.295357042154533
ukb-b-969	ukb-b-17670	462933	rs1028455	-0.004729915	0.00203358055307764	0.020023621292155
ukb-b-969	ukb-b-17670	462933	rs10984444	-0.00520266	0.00215183875366708	0.0156157766623008
ukb-b-969	ukb-b-17670	462933	rs11776021	-0.005337949	0.0021615947587322	0.0135321094575081
ukb-b-969	ukb-b-17670	462933	rs117799466	-0.005419053	0.00215448117466367	0.0118948840678121
ukb-b-969	ukb-b-17670	462933	rs12203592	-0.005800713	0.00211392705154603	0.00606870054520014
ukb-b-969	ukb-b-17670	462933	rs13251020	-0.005534638	0.00214441233434002	0.00985265983943234
ukb-b-969	ukb-b-17670	462933	rs1368551	-0.005210522	0.00217685631999689	0.0166839693371139
ukb-b-969	ukb-b-17670	462933	rs139577	-0.005143233	0.00214651523514157	0.0165712468426894
ukb-b-969	ukb-b-17670	462933	rs1449390	-0.006089383	0.0020682807941107	0.00323815448658549
ukb-b-969	ukb-b-17670	462933	rs2356278	-0.005822418	0.00209000139384007	0.00533885101267703
ukb-b-969	ukb-b-17670	462933	rs2413639	-0.005451869	0.00215357092304442	0.0113560110233792
ukb-b-969	ukb-b-17670	462933	rs251033	-0.005425497	0.00216561017551606	0.0122348531440974
ukb-b-969	ukb-b-17670	462933	rs2647259	-0.005543151	0.00215008158116684	0.00993417934655309
ukb-b-969	ukb-b-17670	462933	rs3243000	-0.005759389	0.00213633577337458	0.00701946448018892

ukb-b-969	ukb-b-17670	462933	rs35660964	-0.005442806	0.00215334101771839	0.0114841945408202
ukb-b-969	ukb-b-17670	462933	rs3740422	-0.004985115	0.00211887046760215	0.0186365077116575
ukb-b-969	ukb-b-17670	462933	rs4344697	-0.005138509	0.00214930200425964	0.0168126075767667
ukb-b-969	ukb-b-17670	462933	rs61083878	-0.004829241	0.00205805893382228	0.0189505148262472
ukb-b-969	ukb-b-17670	462933	rs62379243	-0.004979127	0.00211187909197729	0.0183897183998348
ukb-b-969	ukb-b-17670	462933	rs627685	-0.005021456	0.00213059577111141	0.0184315962275583
ukb-b-969	ukb-b-17670	462933	rs644799	-0.005094033	0.00216228666471633	0.0184801416838589
ukb-b-969	ukb-b-17670	462933	rs7029718	-0.0053099	0.00216047701894487	0.0139812756255706
ukb-b-969	ukb-b-17670	462933	rs7191618	-0.004740135	0.0020507457297852	0.0208096506873054
ukb-b-969	ukb-b-17670	462933	rs7195043	-0.005542209	0.0021494551981 74	0.0099251780961032
ukb-b-969	ukb-b-17670	462933	rs7560588	-0.005872775	0.00207801946668062	0.0047112585383118
ukb-b-969	ukb-b-17670	462933	rs7578811	-0.005550936	0.0021422805774639	0.00956603043291373
ukb-b-969	ukb-b-17670	462933	rs7587930	-0.00537774	0.00217131586038675	0.0132596130836906
ukb-b-969	ukb-b-17670	462933	rs7773004	-0.005047486	0.00214630006788728	0.0186870815509636
ukb-b-969	ukb-b-17670	462933	rs7852747	-0.005136366	0.0021448509719569	0.0166320270244369
ukb-b-969	ukb-b-17670	462933	rs837065	-0.005781958	0.00211937302354837	0.00636915351846008
ukb-b-969	ukb-b-17670	462933	rs9319835	-0.005072673	0.00212798450266017	0.0171352725577933
ukb-b-969	ukb-b-17670	462933	rs9427232	-0.005920485	0.00205595956164977	0.00398091378203358
ukb-b-969	ukb-b-17670	462933	rs9508711	-0.005873444	0.00208012531334437	0.00474874566770846
ukb-b-969	ukb-b-17670	462933	rs9852529	-0.005375225	0.00218055720173931	0.0136986663704957
ukb-b-969	ukb-b-17670	462933	All	-0.00536955	0.00209625671398753	0.0104222944407857

Supplementary Table 4: Summary of the sensitivity test included in this two-sample mendelian randomization study.

id.exposure	id.outcome	method	Q	Q_df	Q_pval
ukb-b-151	ukb-b-17670	MR Egger	3.18631406195323	7	0.867248305458168
ukb-b-151	ukb-b-17670	Inverse variance weighted	4.09295476247385	8	0.848640824550107
ukb-b-1553	ukb-b-17670	Inverse variance weighted	3.64410183428853	1	0.0562681456084673
ukb-b-3793	ukb-b-17670	MR Egger	0.680499162690897	2	0.711592700334349
ukb-b-3793	ukb-b-17670	Inverse variance weighted	2.27719952135966	3	0.516902940320506
ukb-b-4000	ukb-b-17670	MR Egger	4.37473418524326	4	0.357660209260994
ukb-b-4000	ukb-b-17670	Inverse variance weighted	5.45775491031733	5	0.362600448468195
ukb-b-4077	ukb-b-17670	MR Egger	0.541212682054444	1	0.461930585808951
ukb-b-4077	ukb-b-17670	Inverse variance weighted	0.908561820577684	2	0.634904357026924
ukb-b-4171	ukb-b-17670	MR Egger	14.2735032862047	10	0.160877821935883
ukb-b-4171	ukb-b-17670	Inverse variance weighted	14.8876884562089	11	0.18769656668966
ukb-b-4522	ukb-b-17670	MR Egger	81.4370171522261	64	0.0697382718677133
ukb-b-4522	ukb-b-17670	Inverse variance weighted	81.6944192596955	65	0.0789500444610016
ukb-b-4667	ukb-b-17670	MR Egger	12.3700440566719	14	0.576611772107849
ukb-b-4667	ukb-b-17670	Inverse variance weighted	13.3333816241892	15	0.576564601944017
ukb-b-4710	ukb-b-17670	MR Egger	14.7473068945792	10	0.141549771433282
ukb-b-4710	ukb-b-17670	Inverse variance weighted	15.3449701960996	11	0.16724240133911
ukb-b-4886	ukb-b-17670	MR Egger	20.1828306073039	15	0.165030716143061
ukb-b-4886	ukb-b-17670	Inverse variance weighted	22.4506202537166	16	0.129228366739561
ukb-b-50	ukb-b-176	MR Egger	8.44512763735892	6	0.207271269197748

76	70				
ukb-b-5076	ukb-b-17670	Inverse variance weighted	9.8817324165159	7	0.195375248751586
ukb-b-5192	ukb-b-17670	MR Egger	94.6983966951435	76	0.072060886428682
ukb-b-5192	ukb-b-17670	Inverse variance weighted	95.2011557267001	77	0.0781526894819791
ukb-b-6811	ukb-b-17670	MR Egger	0.460873800341026	1	0.497215884371767
ukb-b-6811	ukb-b-17670	Inverse variance weighted	0.772318930744829	2	0.67966213446284
ukb-b-969	ukb-b-17670	MR Egger	45.3216972476877	32	0.0595259911100793
ukb-b-969	ukb-b-17670	Inverse variance weighted	45.8867148668868	33	0.0672331068071814

Supplementary Table 5: The Mendelian randomization pleiotropy residual sum and outlier (MR-PRESSO) analysis in this two-sample mendelian randomization study.

id.exposure	id.outcome	outcome	exposure	egger_intercept pval	MR-PRESSO pval
ukb-b-151	ukb-b-17670	Non-cancer illness code, self-reported: multiple sclerosis || id:ukb-b-17670	Number of days/week of vigorous physical activity 10+ minutes || id:ukb-b-151	0.372719646	0.8563333
ukb-b-1553	ukb-b-17670	Non-cancer illness code, self-reported: multiple sclerosis || id:ukb-b-17670	Leisure/social activities: Adult education class || id:ukb-b-1553	/	/
ukb-b-3793	ukb-b-17670	Non-cancer illness code, self-reported: multiple sclerosis || id:ukb-b-17670	Time spent driving || id:ukb-b-3793	0.333715696	0.5636667
ukb-b-4000	ukb-b-17670	Non-cancer illness code, self-reported: multiple sclerosis || id:ukb-b-17670	Leisure/social activities: Sports club or gym || id:ukb-b-4000	0.376004065	0.3913333
ukb-b-4077	ukb-b-17670	Non-cancer illness code, self-reported: multiple sclerosis || id:ukb-b-17670	Leisure/social activities: Other group activity || id:ukb-b-4077	0.653113617	/
ukb-b-4171	ukb-b-17670	Non-cancer illness code, self-reported: multiple sclerosis || id:ukb-b-17670	Leisure/social activities: Pub or social club || id:ukb-b-4171	0.526647195	0.214
ukb-b-4522	ukb-b-17670	Non-cancer illness code, self-reported: multiple sclerosis || id:ukb-b-17670	Time spent using computer || id:ukb-b-4522	0.654399971	0.08533333
ukb-b-4667	ukb-b-17670	Non-cancer illness code, self-reported: multiple sclerosis || id:ukb-b-17670	Leisure/social activities: Religious group || id:ukb-b-4667	0.343005573	0.562
ukb-b-4710	ukb-b-17670	Non-cancer illness code, self-reported: multiple sclerosis || id:ukb-b-17670	Number of days/week of moderate physical activity 10+ minutes || id:ukb-b-4710	0.538678787	0.172
ukb-b-4886	ukb-b-17670	Non-cancer illness code, self-reported: multiple sclerosis ||	Number of days/week walked 10+ minutes || id:ukb-b-4886	0.21381087	0.1256667

		id:ukb-b-17670			
ukb-b-5076	ukb-b-17670	Non-cancer illness code, self-reported: multiple sclerosis || id:ukb-b-17670	Leisure/social activities: None of the above || id:ukb-b-5076	0.351354038	0.2206667
ukb-b-5192	ukb-b-17670	Non-cancer illness code, self-reported: multiple sclerosis || id:ukb-b-17670	Time spent watching television (TV) || id:ukb-b-5192	0.52720117	0.07
ukb-b-6811	ukb-b-17670	Non-cancer illness code, self-reported: multiple sclerosis || id:ukb-b-17670	Time spent outdoors in winter || id:ukb-b-6811	0.675947853	/
ukb-b-969	ukb-b-17670	Non-cancer illness code, self-reported: multiple sclerosis || id:ukb-b-17670	Time spend outdoors in summer || id:ukb-b-969	0.532124547	0.07933333
